# FALCON: Factory Automation Lab for Cybersecurity & Operational Networks

**DOI:** 10.3390/s26216951

**Published:** 2026-11-03

**Authors:** Mehmet Yavuz Yağcı

**Affiliations:** Department of Computer Engineering, Istanbul University-Cerrahpaşa, 34320 Istanbul, Turkey; myavuz.yagci@iuc.edu.tr

**Keywords:** industrial control systems, SCADA, cyber-physical security, ICS testbed, physical process testbed, attack impact analysis

## Abstract

**Highlights:**

**What are the main findings?**

- FALCON integrates a real, multistage manufacturing process with PLC control, HMI/SCADA supervision, and segmented IT–OT networks in a unified cybersecurity testbed.
- A two-level security analysis links network attacks and physical interventions to generalized process impacts and corresponding observable indicators in FALCON.

**What are the implications of the main findings?**

- The integrated architecture supports investigation of attack consequences for process continuity, process integrity, product quality, and operational safety.
- Generalized impact categories support adaptation of the analysis to other ICS/OT environments, while comparisons of design requirements and attack coverage inform testbed design and evaluation.

**Abstract:**

Industrial Control Systems (ICSs) are increasingly evolving from isolated infrastructures into connected platforms due to the integration of information technology, industrial communication networks, and data-driven monitoring services. Although this transformation improves operational visibility and efficiency, it also expands the attack surface of ICS environments. Conventional vulnerability assessment and penetration testing methods are often unsuitable for operational ICSs because of safety, availability, and cost constraints. Similarly, intrusion detection and mitigation mechanisms require realistic attack data for development and validation, but collecting such data from running industrial systems is risky and impractical. Realistic ICS testbeds provide controlled environments for analyzing cyber-attacks, observing their consequences, and validating security mechanisms without disrupting operational facilities. This study presents FALCON (Factory Automation Lab for Cybersecurity & Operational Networks), a cyber-physical ICS testbed that integrates a real physical production process, PLC-based control, HMI/SCADA supervision, monitoring, logging capabilities, and IT–OT network segmentation. The testbed is designed to represent key functions commonly found in industrial production systems, including sensing, actuation, robotic transfer, quality control, labeling, and sequential process execution. FALCON supports both network-oriented and process-oriented attack scenarios within a controlled environment. Its layered architecture enables attacks to be initiated from different system points and analyzed across communication, control, monitoring, and physical process layers. The proposed testbed was evaluated in terms of design requirements and attack scenario coverage. It also supports a broad set of attack scenarios and enables the observation of immediate or delayed physical consequences.

## 1. Introduction

Industrial Control Systems (ICSs) are essential infrastructures for monitoring and controlling physical processes in industrial environments. They are widely used in sectors such as energy, manufacturing, water treatment, transportation, oil and gas, and chemical production. An ICS typically includes programmable logic controllers (PLCs), supervisory control and data acquisition (SCADA) systems, human–machine interfaces (HMIs), sensors, actuators, and industrial communication networks [1].

Historically, ICSs were deployed as isolated infrastructures separated from enterprise networks and external communication channels. However, the increasing integration of IIoT technologies, remote maintenance services, cloud platforms, and data-driven monitoring systems has significantly expanded connectivity between industrial and enterprise environments. While these developments improve operational efficiency and visibility, they also enlarge the attack surface and create new cyber-physical dependencies [2]. Consequently, unlike traditional IT attacks that primarily result in data loss or service disruption, attacks on ICSs can directly influence physical processes through manipulated control commands, altered sensor readings, or disrupted PLC–HMI communications, potentially causing unsafe operating conditions, equipment malfunction, and production losses.

Several real-world incidents have highlighted the cyber-physical nature of ICS threats. Stuxnet demonstrated that malware could manipulate PLC-controlled processes and cause physical damage [3]. Havex/Oldrea showed how malware could perform ICS-focused reconnaissance [4], while the BlackEnergy attacks against the Ukrainian power grid illustrated the potential for large-scale service disruption [5]. Industroyer further revealed the risks posed by malware specifically designed for industrial communication protocols and operational processes [6]. Accordingly, the analysis of ICS threats requires experimental platforms that can reproduce industrial processes, support repeatable attack scenarios, and reveal the relationship between cyber events and physical consequences. In this context, intrusion detection systems (IDSs) play a critical role in identifying abnormal network behavior, unauthorized control actions, and process-related deviations before they lead to operational disruption [7,8,9]. However, the development and validation of effective IDSs require realistic traffic traces, representative attack scenarios, and observable process responses, which are difficult to obtain from operational ICS environments.

ICS testbeds provide controlled experimental environments for analyzing vulnerabilities, reproducing attack scenarios, collecting realistic data, and validating defense mechanisms without affecting operational facilities. Existing ICS testbeds can be implemented in different forms, including software-based, digital-twin-based, semi-physical, and physical [10]. Software-based and simulation testbeds offer low cost, scalability, and repeatability, but they may abstract physical process behavior. Digital twin-based testbeds enable virtual representation and synchronization of industrial processes, but their realism depends on model accuracy and data integration. Semi-physical and hardware-in-the-loop testbeds combine real control components with simulated or partially physical processes, providing a balance between flexibility and realism. Physical testbeds offer the highest process fidelity by using real sensors, actuators, controllers, and industrial devices; however, they are often costly, less scalable, and more difficult to modify. Therefore, an effective ICS testbed should satisfy key design requirements such as fidelity, modularity, reproducibility, scalability, interoperability, monitoring and logging capability, IT–OT visibility, isolation, and flexibility for diverse attack scenarios.

This study presents FALCON (Factory Automation Lab for Cybersecurity & Operational Networks), a cyber-physical ICS/SCADA testbed designed as a scaled but functional representation of an industrial production environment. FALCON integrates a real physical production process with PLC-based control, HMI/SCADA supervision, sensor and actuator layers, robotic transfer mechanisms, station-based quality control operations, monitoring and logging capabilities, and layered IT–OT network segmentation. This structure enables cyber-attacks to be evaluated not only at the network and controller levels, but also through their observable effects on material flow, process sequencing, robotic movement, operator visibility, and station-based production behavior.

The main contributions of this study are as follows:

- Design and implementation of a physical ICS/SCADA testbed integrating a multi-stage production process with industrial control and supervision.
- Structured analysis of network- and process-based attacks, linking relevant MITRE ATT&CK for ICS techniques to expected process impacts and FALCON-specific observable indicators.
- Experimental evaluation of two representative attacks through comparisons of recorded process states under normal and attack conditions.

The remainder of this paper is organized as follows. Section 2 reviews related studies on ICS testbeds and their approaches to cybersecurity evaluation. Section 3 presents the design requirements and objectives considered in the development of FALCON. Section 4 describes the proposed testbed architecture, including its physical processes, industrial control components, and network infrastructure. Section 5 presents the security analysis of FALCON, focusing on network-oriented and process-oriented attack scenarios. Section 6 discusses the results, including comparisons with existing testbeds and experimental validation of selected attack scenarios. Finally, Section 7 concludes the paper and outlines directions for future research.

## 2. Related Work

### 2.1. Digital Twin

Digital twin technology aims to reduce the cost of cybersecurity analyses and prevent operational disruptions by enabling the dynamic and synchronous modelling of physical assets or processes in virtual environments [11]. This approach has been widely adopted, particularly for creating virtual replicas to evaluate the cyber resilience of production and manufacturing systems [12]. In this context, Olivares-Rojas et al. [13] developed a digital twin model to assess cybersecurity vulnerabilities in advanced metering infrastructures (AMI) within smart home environments and performed vulnerability scanning in a virtual environment. Similarly, Masi et al. [14] designed a digital twin of an industrial control system based on cybersecurity requirements; using this model, they derived potential attack paths and analysed the corresponding defence mechanisms.

The security risks introduced by digital twins have also been discussed in the literature. Holmes et al. [15] and Repetto [16] emphasized that digital twins may themselves constitute an attack surface and that adversaries could exploit these virtual environments to gather intelligence about the target physical infrastructure. In addition, the possibility that a virtual model may not fully represent all critical threats or extreme failure conditions of the physical system has been identified as an important limitation that should be considered during the validation of digital twin-based security testing. In this regard, refs. [17,18] developed a fully virtualized digital twin of the physical EPIC power system testbed, referred to as EPICTWIN. By modelling network interactions over MQTT, IEC 61850 MMS, and GOOSE protocols, EPICTWIN provides a scenario-based environment suitable for threat analysis and vulnerability assessment. Overall, digital twin studies generally focus on high-level protocol and data-flow modelling at the architectural level, whereas the fidelity of physical process dynamics and the realism of the network layer remain key evaluation criteria.

### 2.2. Industrial Control System Testbeds

Cybersecurity research on industrial control systems cannot typically be conducted on live operational systems due to the risk of service disruption, safety implications, and high implementation costs. Therefore, researchers have developed testbeds to investigate cyber-physical security issues under controlled, repeatable, and observable experimental conditions. In the literature, ICS testbed studies are commonly classified into three main architectural categories according to the nature of their constituent components: software-based, semi-physical, and physical testbeds [19].

#### 2.2.1. Software-Based Testbeds

Software-based testbeds provide low-cost and highly flexible experimental environments by simulating control components, network infrastructure, and human–machine interfaces on virtual machines or containers. Queirozet al. [20] proposed SCADASim, a framework built on the OMNET++ event-based simulation engine, in which basic classes were defined for RTU, PLC, and MTU components, and DDoS and MITM attack scenarios were evaluated. Although the architecture was designed to be extensible, the absence of a Hardware-in-the-Loop (HIL) interface and a dedicated network traffic logging component was reported as a limitation for comprehensive evaluation.

Antonioli et al. [21] developed MiniCPS, a Python- and Mininet-based tool that enables the virtual simulation of PLCs, HMIs, and industrial protocols such as Modbus. The tool was used to model network and control interactions in water treatment systems. While its flexible structure allows different control scenarios to be rapidly adapted, the limitations imposed by running Mininet on a single host and the lack of a comprehensive logging mechanism restrict its applicability for network-based attack analysis.

Morris et al. [22] developed a virtual gas pipeline testbed by integrating network, PLC, HMI, and HIL components within a fully software-based environment. The study analysed sensor attacks using command cycles that randomized system states. However, the support for only serial Modbus communication and the absence of realistic network traffic simulation were identified as limitations for network-layer security testing.

Formby et al. [23] introduced the GRFICS platform, which integrates 3D visualization, HIL simulation of a chemical process, and an OpenPLC code generator. The platform was used to validate Modbus buffer overflow and command injection attacks. Nevertheless, its education-oriented design and dependence on a predefined chemical process scenario limit its adaptability to different industrial domains.

To improve the scalability of virtual environments, Maynard et al. [24] proposed a modular SCADA network framework deployed on virtual machines. By using OpenPLC and Vagrant-based configurations, the framework virtually models OT protocol communication between HMI and RTU components. This architecture is particularly notable for its cost-effectiveness and ease of replication in both local and cloud-based environments.

Software-based testbeds are commonly used for protocol-level vulnerability assessment, network traffic analysis, and machine learning-based attack detection scenarios. However, due to the inherent limitations of virtual simulation, physical process dynamics and real-time latency-related metrics are generally evaluated indirectly rather than through direct interaction with real industrial processes.

#### 2.2.2. Semi-Physical Testbeds

Semi-physical, or hybrid, testbeds aim to establish a balance between cost and realism by combining real control devices, such as PLCs and RTUs, with virtualized physical process simulations, including Hardware-in-the-Loop (HIL) and Software-in-the-Loop (SIL) models, or network emulation environments. Gao et al. [25] developed the EPS-ICS testbed by integrating a Matlab/Simulink-based HIL mathematical model with real PLCs and Emulab/PlanetLab-based network emulation. Although the architecture primarily focused on analysing PLC vulnerabilities, the absence of real network traffic and the time-consuming nature of hardware integration were identified as limiting factors for network-based security evaluation. Similarly, Koganti et al. [26] simulated PLC and HIL components in software while constructing the network layer using real physical switches. This approach provides high flexibility through the modification of simulation scripts; however, the lack of a comprehensive HMI application limits its suitability for operator interaction scenarios.

Faramondi et al. [27] integrated miniCPS-based virtual ICS components with real HIL tank and pipeline systems for a water distribution scenario. Owing to its multi-controller structure, the testbed enabled high-fidelity physical attack scenarios and resulted in the publication of a dataset containing both normal and attack-state network data. Nevertheless, the use of Mininet for network simulation was considered an architectural limitation, as it makes it difficult to evaluate realistic network latency and packet loss scenarios.

The capability of hybrid architectures to examine IT–OT interactions has also been addressed in studies that combine physical and virtual components. Mallouhi et al. [28] proposed an architecture focused on real-time event simulation through the integration of physical and virtual elements. Koutsandria et al. [29] developed a testbed by integrating real PLCs and a Simulink-based OT simulation with a Raspberry Pi-based network tap infrastructure, enabling the monitoring of network traffic statistics and packet-level changes. Although the study demonstrated the feasibility of implementing attack scenarios, the non-public availability of the source code limited reproducibility.

Keliriset al. [30] combined hardware and software components within a hybrid architecture to evaluate machine learning-based defence mechanisms. The study performed vulnerability and impact analysis; however, it did not cover network-layer attacks such as ARP poisoning and packet sniffing. Ghaleb et al. [31] introduced SCADA-SST as a lightweight and scalable hybrid platform that supports communication between virtual and real devices. The platform was used to evaluate DoS/DDoS attack scenarios and traffic capture metrics. Xie et al. [32] developed the VTET testbed by combining a virtualization infrastructure with the Tennessee Eastman Process simulation. Although the testbed supports multiple ICS protocols, the absence of network-based penetration scenarios narrows its evaluation scope.

Semi-physical testbeds commonly focus on the stability of control algorithms, latency between PLC software and physical process simulations, and the effects of multi-stage attacks at the IT–OT boundary.

#### 2.2.3. Physical Testbeds

Physical testbeds provide the highest level of realism and fidelity by establishing full-scale or scaled-down hardware environments that include real industrial actuators, sensors, PLCs, and SCADA systems. Mathur et al. [33] developed the SWaT testbed by replicating a six-stage water treatment process, in which each stage is controlled by autonomous real PLCs, enabling the evaluation of multi-layer cyber-physical attack scenarios.

The National SCADA Testbed [34] simulated a 61-mile, 138-kV power distribution network at field scale and evaluated the cyber resilience of energy control systems through security scenarios, advanced visualization, and modelling tools. However, the large physical infrastructure and substantial budget requirements make the testbed difficult to reproduce for most researchers.

As a low-cost physical alternative, Alves et al. [35] developed the OpenPLC platform and implemented open-source, low-cost PLCs through a modular and simplified architecture, enabling the evaluation of hardware-based control scenarios. Nevertheless, the need for hardware expertise, the decreasing availability of compatible components over time, and the limited architectural scope create challenges for extensibility and long-term evaluation. Similarly, Sauer et al. [36] introduced LICSTER, an open-source ICS testbed designed with an approximate budget of 500 euros, providing an accessible physical control environment for students and researchers. However, the requirement for physical hardware assembly prevents the testbed from being shared as a purely software-based artefact and increases the level of technical expertise needed for deployment.

Adepu et al. [18] developed the EPIC testbed by physically replicating a smart grid architecture using real PLCs and the IEC 61850 protocol. The platform was used to evaluate attack scenarios with physical consequences, such as false data injection and power outage events. The Smart Grid Test Bed developed by Idaho National Laboratory (2017) also provides a full-scale smart grid replication and demonstrates scalability in the transition from small-scale pilot systems to real-world infrastructure.

Physical testbeds are commonly regarded as the gold standard for impact analysis and physical security validation because they allow the tangible physical consequences of attacks, such as pressure variations, motor vibration, and valve position deviations, to be directly measured. However, high cost, complex deployment, maintenance requirements, and isolation-related risks often limit the availability of such testbeds to national laboratories or specialized research centers.

Table 1 provides a comparative overview of the reviewed studies, highlighting their implementation approaches, application domains, and the scope of their cybersecurity evaluations.

The reviewed approaches highlight a trade-off between experimental accessibility and the faithful representation of industrial cyber-physical behavior. Digital twins and software-based testbeds support flexible security testing, but the representation of physical dynamics and communication behavior depends on the underlying models and their validation. Semi-physical testbeds combine real and simulated components, while some aspects of system behavior remain simulation dependent. Physical testbeds enable direct measurement of attack consequences, but their construction, operation, and maintenance can impose substantial resource requirements. These trade-offs motivate a laboratory-scale manufacturing environment in which real control devices, supervisory systems, network communications, and physical operations can be examined together. The specific gap addressed by FALCON concerns tracing how network- and process-based attacks affect interdependent manufacturing stages, linking attack actions to observable changes in control behavior, material flow, robotic operations, and downstream production outcomes.

## 3. Design Requirements and Objectives

The design of an ICS/SCADA cybersecurity testbed requires a clear definition of the capabilities that make the environment suitable for security experimentation [19,37,38]. In this study, eleven design requirements are considered: cost-effectiveness, monitoring and logging, flexibility, reproducibility, high fidelity, scalability, granularity, isolation, usability, diversity, and interoperability. These requirements define the expected architectural and operational qualities of the testbed, including implementation cost, process observability, experimental realism, repeatability, safe attack execution, ease of use, system expansion, and the integration of heterogeneous cyber and physical components. The following paragraphs define each requirement and explain how it is addressed in the FALCON testbed architecture.

**Cost-effectiveness:** Cost-effectiveness refers to the ability to develop testbed designs and scenarios that are aligned with the research objectives within an appropriate budget [39,40]. The main objective is to achieve the targeted design and experimental scenarios at substantially lower cost than would be required for real industrial systems. To reduce costs compared with real-world deployments, some components and services need to be implemented at a smaller scale, while others may be simulated [41]. Therefore, testbed development requires an objective-driven balance between implementation cost and the degree to which the system reflects real operational conditions.

**Monitoring and logging:** Monitoring and logging refer to the observation, recording, and retention of event logs for subsequent analysis, as well as the optimization of these processes [42,43]. Event logging typically relies on multiple data sources, including operational process monitoring, data collected from traffic monitoring systems, and logs obtained from operating system-level logging tools [44]. This capability can be further enhanced through automated and fine-grained data flows that capture relevant data sources and communication paths, enabling undesired effects on process functionality to be analyzed more effectively [45].

**Flexibility:** Flexibility refers to the ability to redefine alternative use-case scenarios within a simulation environment or testbed [44,46]. The scenarios defined in such environments interact with monitoring and control components, as well as with other system elements, through data exchange. Therefore, flexibility is closely associated with controllability and observability [47]. A representative example of flexibility is a system that is initially designed to analyze security vulnerabilities but can later be reconfigured to evaluate the effectiveness of security countermeasures.

**Reproducibility:** Reproducibility refers to the ability of systems with the same design to produce comparable outputs. Testbed designs should have similar configurations and should generate identical or statistically consistent results when similar experiments are conducted [29,39]. One way to improve reproducibility is to report intermediate outcomes observed during the physical process and to enable cross-checking across experimental runs. In addition, the testbed design, process configurations, and security scenarios should be documented comprehensively [42].

**High fidelity:** High fidelity refers to the extent to which simulations executed within a process and the resulting outputs correspond to observations in the real world [39,48]. This concept covers both hardware and software technologies, together with their functions and roles, and indicates how representative a testbed is in comparison with the actual system. The degree of fidelity is generally determined by the selected simulation approach, which may involve software-based simulation (SBS), semi-physical simulation (SPS), physical simulation (PS), or a combination of these approaches. Physical simulation is typically considered to provide the highest level of fidelity, whereas fully software-based simulations are generally associated with the lowest level of fidelity [46].

**Scalability:** Scalability refers to the ability to increase the size and functionality of the testbed architecture [43,49]. This capability can be defined as the possibility of adding new field components to the existing structure, relocating components into subsystems, extending the network architecture, and integrating additional IT devices. In this way, new capabilities and functions can be introduced by expanding the existing system without requiring a complete redesign. Scalability can also be improved by simulating field devices or subsystems when physical expansion is not feasible.

**Granularity:** Granularity refers to the extent to which details are represented in real-world modelling. The level of detail included in a model directly affects the consistency of the data obtained from the process and the range of scenarios that can be tested [50]. As the level of detail increases, the time, cost, and effort required for implementation also increase; however, the resulting model becomes more precise and representative. One of the key factors affecting granularity is the use of real devices from the reference system being modelled. For a model designed to investigate network-based attacks, real industrial devices and protocols should be used so that the components communicate in a manner that closely resembles, or is identical to, the network traffic observed in real ICS environments.

**Isolation:** Isolation refers to the ability to develop and execute cybersecurity scenarios and attack vectors within a secure testbed or model environment that is separated from external networks, thereby minimizing any potential impact on the outside network [51]. This capability can be achieved through network segmentation that separates the testbed network from the institutional or enterprise network.

**Usability:** Usability refers to the ease with which operators with a reasonable level of skill can use a test environment. This capability minimizes the risk of incorrect use of the simulation. Usability can be achieved by adopting design principles that support human-centred user interfaces and by developing appropriate architectural structures [52]. For example, the use of virtualization and VLANs can simplify the integration of testbed components within the Control Group (CG) section of ICS architectures. In the security context, weak or insufficient usability may lead to confusion, frustration, and incorrect or inadequate configuration of security scenarios, tools, and functions. As a result, users may unintentionally weaken the intended security features, ultimately limiting the effectiveness of the system and the model [53].

**Diversity:** Diversity refers to the ability of a testbed to incorporate various components without compromising its scalability. Testbeds with a broad architectural scope should be capable of representing different configurations [42]. This capability reflects the heterogeneous nature of real-world systems by including components from different vendors, models, and protocols. Such heterogeneity strengthens the representativeness of the testbed and contributes to high fidelity by enabling the environment to reflect real-world problems and system behaviors more accurately.

**Interoperability:** Interoperability refers to the ability of testbed components, such as the physical process, network infrastructure, security mechanisms, and physical devices, to interface, communicate, and exchange information with each other in order to achieve the intended objectives [41]. This capability is particularly important in physical and semi-physical testbeds, where communication must be established between physical components and software-based components, as well as between subsystems running on virtual machine servers.

## 4. FALCON Testbed Design

### 4.1. Design Architecture

**Figure 1:**
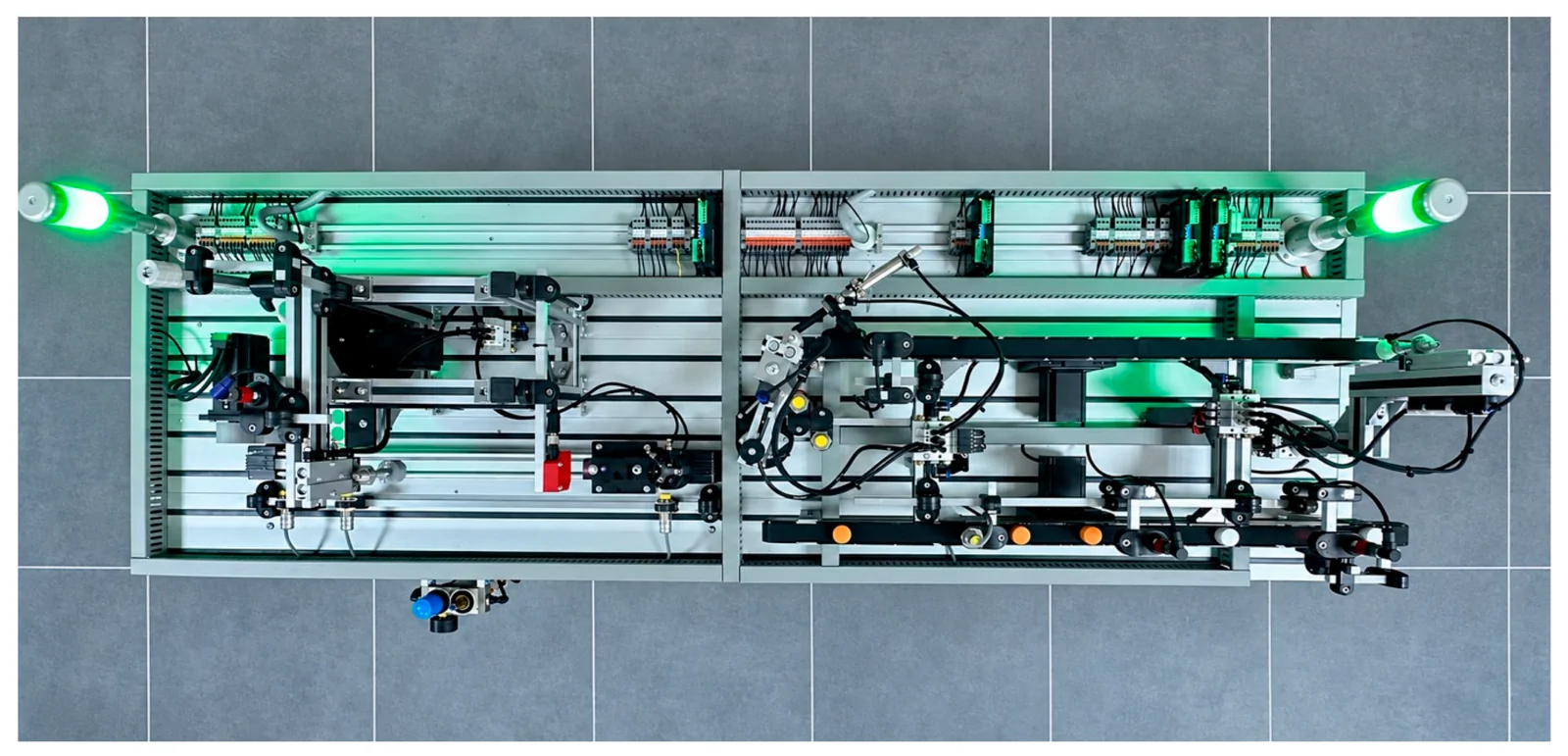
Overall physical layout of the developed testbed.

The testbed was designed as a scaled yet functional representation of an industrial production line, following a modular, layered, and extensible architectural approach. The architecture consists of two parallel conveyor lines, pneumatic and servo-based robotic arms, a single-axis linear positioning system, sensors, actuators, and industrial control units. These components collectively provide a small-scale but operationally integrated representation of material handling, sorting, robotic transfer, quality control, and station-based processing mechanisms typically encountered in industrial production systems. An overall view of the testbed is presented in Figure 1, showing the physical arrangement of its main production components.

The first production line, Conveyor-1, was designed to operate autonomously and represents unmanned production lines. This conveyor is 80 cm long and 2 cm wide and is driven by a stepper motor operating at 40 rpm. The sensors positioned along Conveyor-1 are distributed at predefined intervals to monitor different stages of the production flow. These sensors are responsible for identifying the raw material type, counting the products, and triggering the end-of-line transfer operation. This structure enables the laboratory-scale representation of automatic sensing, monitoring, and control functions used in industrial production lines.

The pneumatic robotic arm located at the end of Conveyor-1, referred to as the P-Robot, provides the physical connection between the two production lines. This robotic arm has dimensions of 35 cm × 15 cm, operates at 6 bar pressure, and provides an angular motion range of 0–180°. Through its vacuum-based holding mechanism and limited vertical motion capability, the P-Robot enables objects to be transferred between conveyor lines in a safe, repeatable, and controlled manner. This component represents the pick-and-place operations commonly used in industrial automation systems.

The second production line, Conveyor-2, has the same physical dimensions as Conveyor-1 and is positioned in parallel with it. However, Conveyor-2 differs from Conveyor-1 in terms of automation level and represents a semi-automated production line that allows human intervention. Unlike Conveyor-1, this line does not include distributed sensors for continuous process monitoring; instead, a single sensor is located at the end of the conveyor for product verification. This design choice enables the experimental representation of operator involvement and limited automation in human-supported production processes.

The servo-based robotic arm located at the end of Conveyor-2, referred to as the E-Robot, is the main component responsible for precise handling and redirection operations. This robotic arm has dimensions of 40 cm × 20 cm, provides a motion range of 0–270°, and operates with a vacuum-based holding mechanism. The E-Robot enables bidirectional material transfer between the conveyor line and the linear axis system, thereby connecting the conveyor-based production flow to the station-based processing stage.

The linear axis system was designed for high-precision positioning and sequential station-based operations. This system is 70 cm long, moves back and forth along a single axis, and is controlled by stepper motors. Unlike the conveyor systems, which operate based on continuous material transport, the linear axis system positions the product between predefined processing stations. A part holder, quality control unit, labeling unit, and stamping unit are positioned on the linear axis. This configuration provides a scaled representation of station-based processing and quality assurance architectures used in industrial production lines.

FALCON architecture integrates autonomous production, human-supported processing, robotic transfer, station-based quality control, and digital process integration within a single modular experimental infrastructure. This structure enables industrial process behavior and cyber-physical security scenarios to be investigated under controlled and repeatable laboratory conditions.

### 4.2. Physical Process

**Figure 2:**
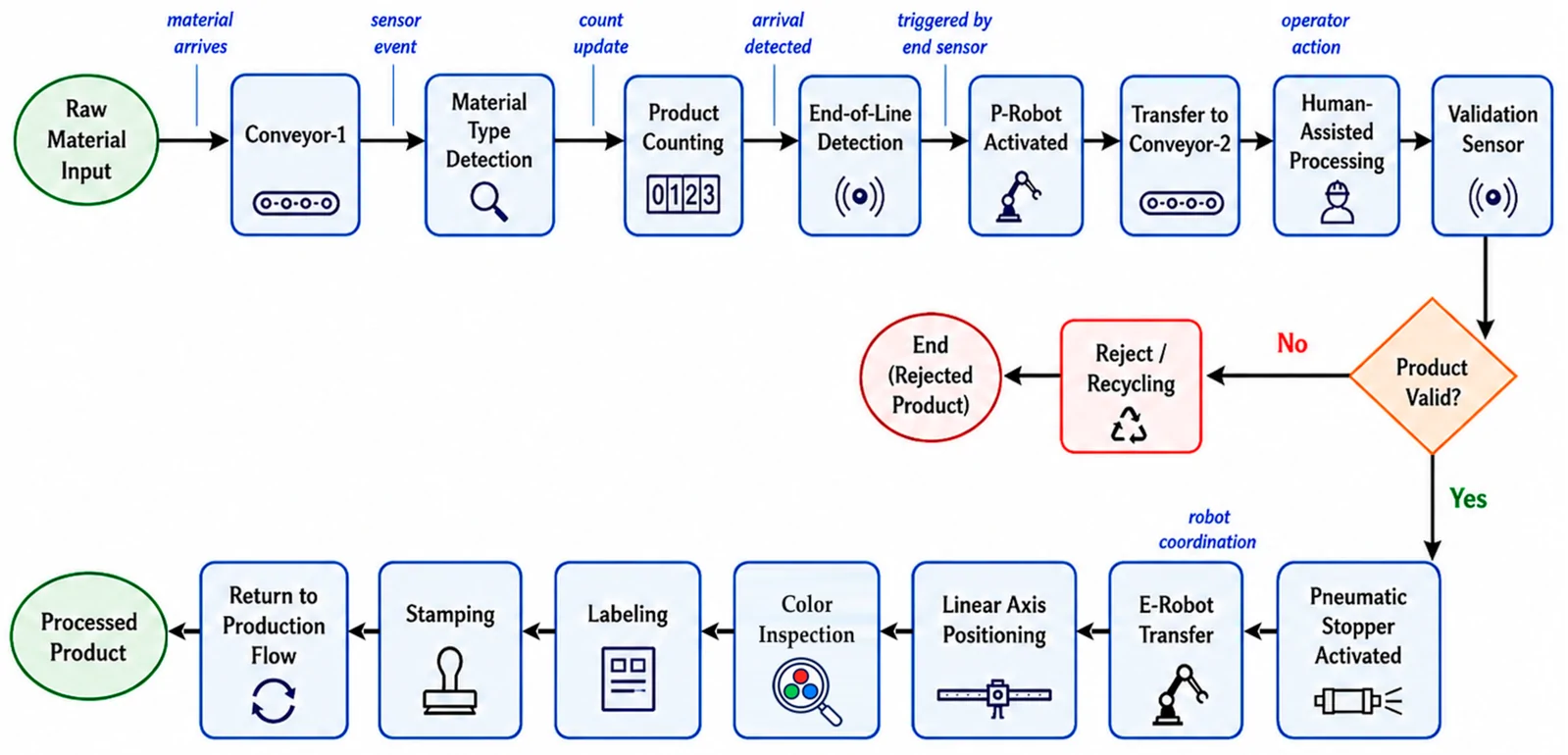
Physical process workflow.

The physical process implemented in the testbed was designed as a sequential and event-driven production workflow, starting from the entry of raw materials into the system and continuing through quality control, labeling, and stamping operations. The overall process flow and the interactions between the physical components are presented in Figure 2. The workflow was modeled to represent the material flow, sensing, decision-making, robotic transfer, and quality-based separation mechanisms commonly observed in industrial production lines.

The process begins with the introduction of plastic and metal raw materials into the system via Conveyor-1. As the raw materials move along the conveyor, the material type is first detected, and the number of items within the production flow is subsequently counted. This stage provides an experimental representation of automatic classification and inventory-tracking mechanisms used in industrial production environments. The sensor located at the end of Conveyor-1 detects when the raw material reaches the transfer point and triggers the activation of the pneumatic robotic arm.

The P-Robot grasps the incoming raw material and transfers it to Conveyor-2. This operation represents a scaled implementation of automated intermediate transfer mechanisms commonly used in production lines. As the material moves along Conveyor-2, it is processed with human intervention and converted into a product. This stage represents the role of the operator in semi-automated production processes and enables the testbed to incorporate human-in-the-loop behavior within the physical workflow.

The verification sensor located at the end of Conveyor-2 determines whether the product is suitable for transfer to the next processing stage. If the product satisfies the verification criteria, the pneumatic stopper mechanism is activated to hold the product in position. Otherwise, the product is directed out of the production flow and transferred to the recycling process.

Validated products are picked up by the servo-based robotic arm, referred to as the E-Robot, and placed into the part holder on the linear axis system. The linear axis system then moves the product sequentially through the quality control, labeling, and stamping stations, thereby executing the station-based processing stage of the workflow. In the first stage, color inspection is performed; products that pass this inspection are transferred to the labeling station, where a quality label is applied. The product is then moved to the stamping station, completing the physical processing sequence.

After successfully completing the quality control and marking operations, the product is retrieved by the E-Robot and reintegrated into the physical production line. This cycle establishes a continuous production flow within the testbed while also creating a synchronized data and event flow between the physical process and the digital systems.

Overall, this structure integrates event-driven control, deterministic production flow, human–machine interaction, robotic transfer, and quality-oriented decision-making mechanisms within a single experimental environment. As a result, the testbed provides not only a platform for examining physical production processes but also a suitable experimental infrastructure for observing and analyzing cyber-physical effects that may occur within industrial automation systems.

### 4.3. ICS Component

The testbed incorporates a set of industrial control system components that enable the monitoring, control, coordination, and communication of the physical production process. The main ICS components include three programmable logic controllers, one human–machine interface, one SCADA system, a power analyzer for real-time electrical measurements, an industrial Ethernet switch, and complementary IT-side components used for monitoring, data acquisition, and analysis. The testbed uses Delta PLCs DVP-12SE, extensions modules DVP-08SN and DVP08SM, a Kinco HMI GL100E, a Klemsen power analyzer Powys, a Murr industrial Ethernet switch Xelity 8TX, and DeltaView SCADA software. These components are integrated with the physical production modules described in the previous sections, thereby forming a complete cyber-physical industrial automation environment.

The control architecture is distributed across three PLCs, each of which is responsible for a specific functional segment of the production process. This distribution of control responsibilities reflects the modular design philosophy of the testbed and enables each production subsystem to be operated, monitored, and analyzed independently while maintaining coordinated operation at the system level.

PLC-1 is responsible for controlling the processes associated with the servo-based robotic arm, referred to as the E-Robot. It receives the signal indicating that a product has been stopped by the stopper mechanism at the end of Conveyor-2 and coordinates the transfer of the product from Conveyor-2 to the linear axis system. After the product completes the quality control and labeling operations on the linear axis, PLC-1 receives the corresponding ready signal and commands the E-Robot to retrieve the processed product and return it to Conveyor-1. In this manner, PLC-1 acts as the main controller for the bidirectional material transfer between Conveyor-2, the linear processing unit, and Conveyor-1.

PLC-2 is responsible for the operation of the conveyor-based production flow and the pneumatic robotic arm, referred to as the P-Robot. It monitors the sensors located on Conveyor-1 and manages the initial processing of raw materials entering the testbed. Through these sensor signals, PLC-2 identifies the type of raw material, tracks production-related statistics, and controls the movement of materials along Conveyor-1. Once the operations on Conveyor-1 are completed, PLC-2 coordinates the transfer of the material to Conveyor-2 using the P-Robot. It also controls the movement of the product along Conveyor-2 and activates the stopper mechanism when a product of appropriate dimensions reaches the end of the conveyor. At this stage, PLC-2 communicates with PLC-1 to initiate the product transfer from Conveyor-2 to the E-Robot handling area.

PLC-3 is responsible for controlling the linear axis system where quality control, labeling, and stamping operations are performed. After receiving the signal from PLC-1 indicating that the product has been placed on the linear axis, PLC-3 activates the holding mechanism and moves the product to the quality control station. Following the quality inspection step, the product is sequentially transferred to the labeling and stamping units. Once the station-based processing sequence is completed, PLC-3 generates the ready signal required for the E-Robot to retrieve the product from the linear axis. Therefore, PLC-3 functions as the dedicated controller for the station-based processing and quality assurance segment of the testbed.

**Figure 3:**
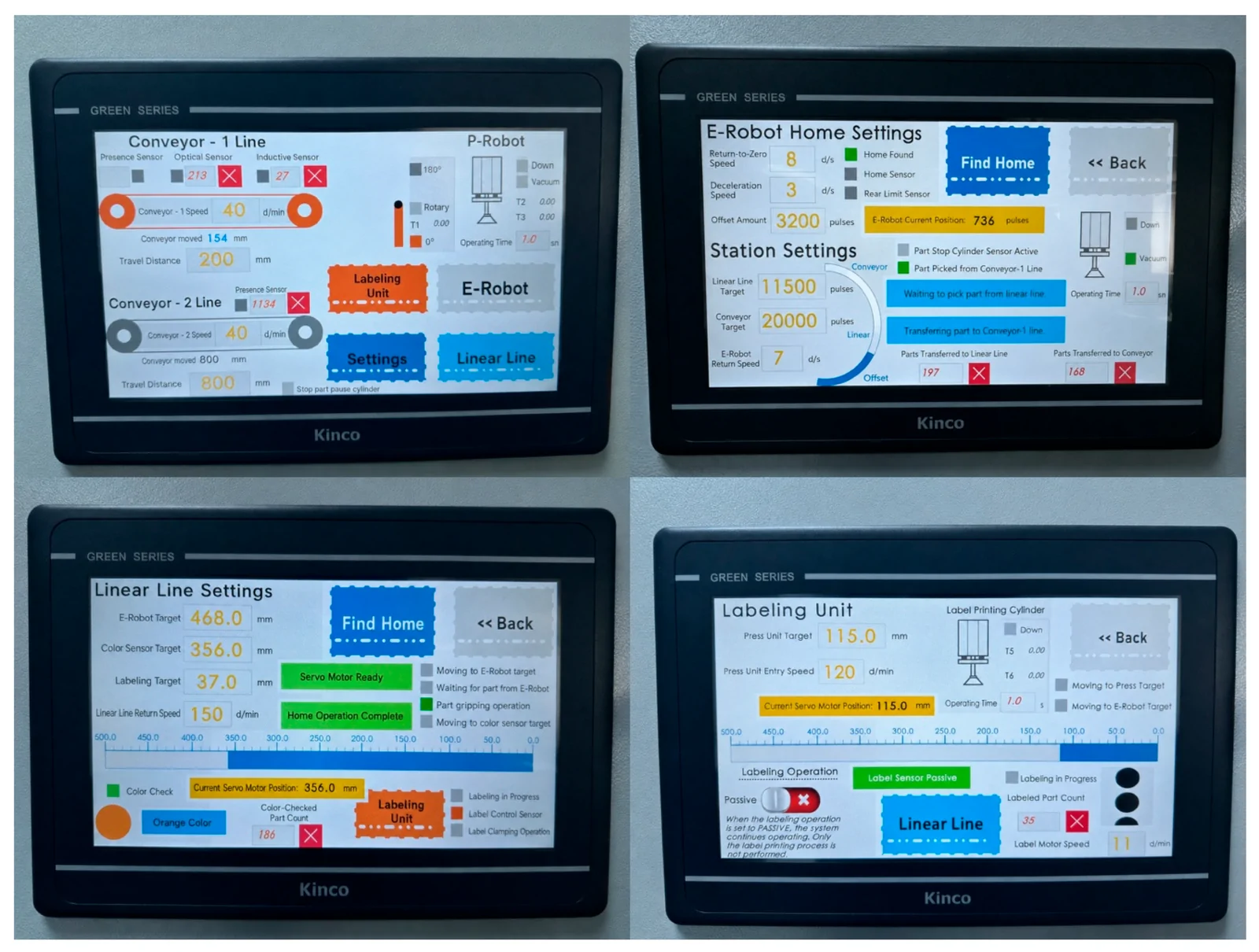
FALCON HMI.

The HMI provides an operator-oriented interface for monitoring field-level data, performing partial control of process parameters, and executing calibration operations required before the production process begins. Through the HMI, operators can observe selected sensor states, actuator conditions, and process parameters while also performing limited supervisory actions. The HMI screen used for field-level monitoring, partial parameter control, and pre-operation calibration is shown in Figure 3. This component supports human–machine interaction within the testbed and enables the investigation of operator involvement in semi-automated production scenarios.

**Figure 4:**
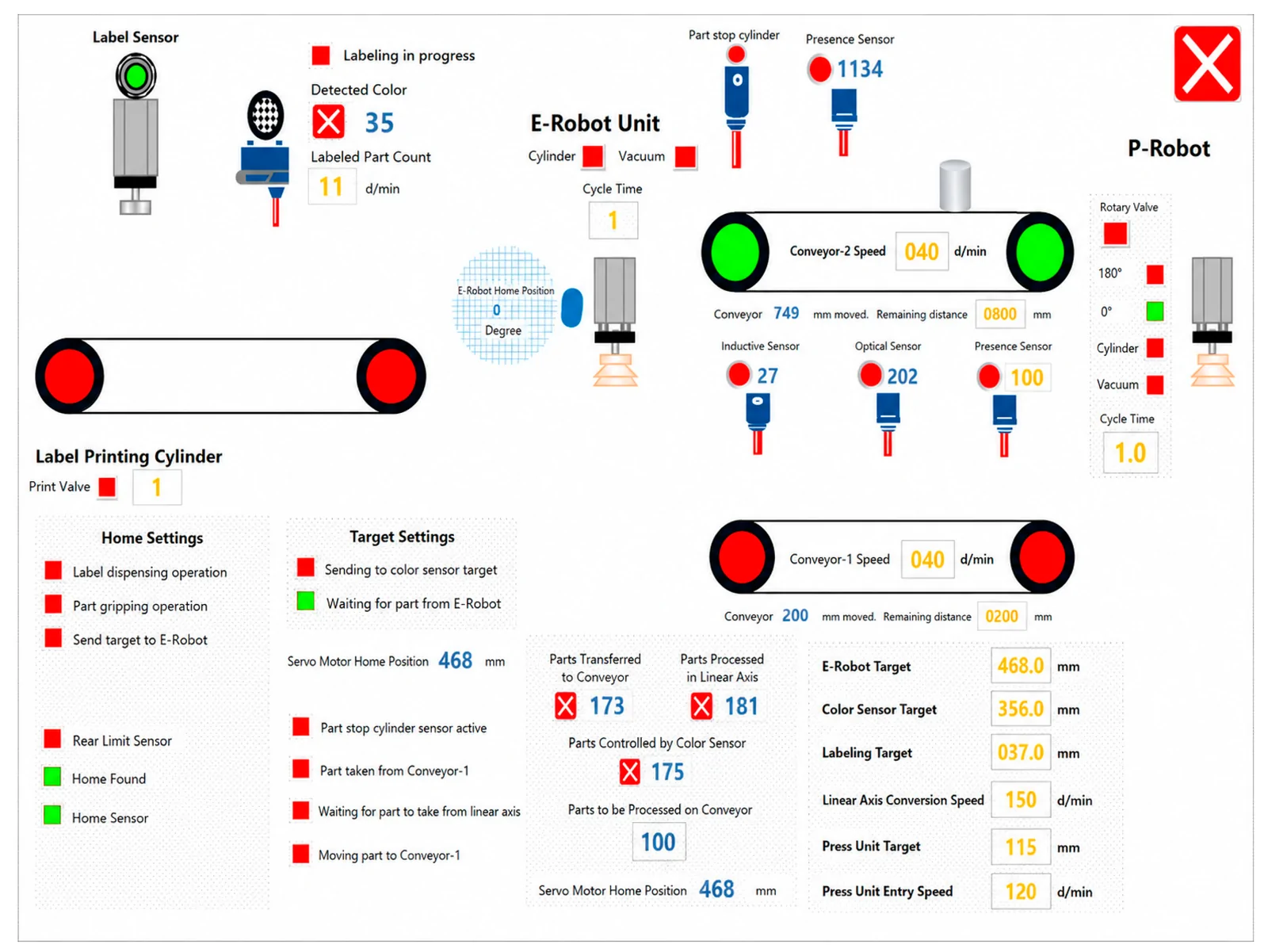
FALCON SCADA.

The SCADA system provides centralized real-time monitoring of the overall testbed operation. It visualizes process changes, component states, production progress, and event information on a single supervisory screen. The SCADA screen that visualizes the overall production flow and real-time process states is presented in Figure 4.

By presenting the entire production flow from raw material entry to final product handling, the SCADA system enables operators and researchers to observe the operational state of the testbed in an integrated manner. This capability is particularly important for cyber-physical security experiments, since deviations in field behavior, controller responses, or process timing can be monitored at the supervisory level.

A power analyzer is integrated into the testbed to monitor the electrical characteristics of the system during operation. It measures the total voltage, current, and instantaneous power consumption of the testbed components. These measurements provide an additional observation channel for evaluating the operational behavior of the system and can be used to correlate physical process states with electrical consumption patterns. In cybersecurity-oriented experiments, such measurements may also support the analysis of abnormal operating conditions caused by process faults or cyber-induced manipulations.

The industrial Ethernet switch forms the core communication infrastructure of the testbed. It enables communication among PLCs, the HMI, the SCADA system, the power analyzer, and the IT-side monitoring and analysis components. Through this network infrastructure, field-level control data, supervisory monitoring information, and measurement data are exchanged across different layers of the testbed. The switch also provides a practical point for traffic monitoring and data collection, allowing industrial communication patterns to be captured without disrupting the normal operation of the production process.

The ICS component provides a distributed and modular control structure in which each PLC manages a dedicated part of the physical process, while the HMI and SCADA system support operator interaction and supervisory monitoring. The integration of the power analyzer and industrial communication infrastructure further extends the testbed from a purely physical automation platform to a cyber-physical experimental environment. This structure enables the synchronized observation of control signals, process states, network communication, and electrical measurements, thereby supporting repeatable experiments for industrial automation and cybersecurity research.

### 4.4. Network

Industrial Control Systems (ICS) commonly rely on IP-based communication protocols such as Modbus TCP, OPC UA, EtherNet/IP, and DNP3 [10,50,54]. Among these, the FALCON testbed adopts Modbus TCP due to its widespread use and extensive coverage in the literature. Modbus was first used to connect industrial devices over serial links. As networks have developed, they have been able to use TCP/IP. This has allowed data to be exchanged across IP-based networks.

**Figure 5:**
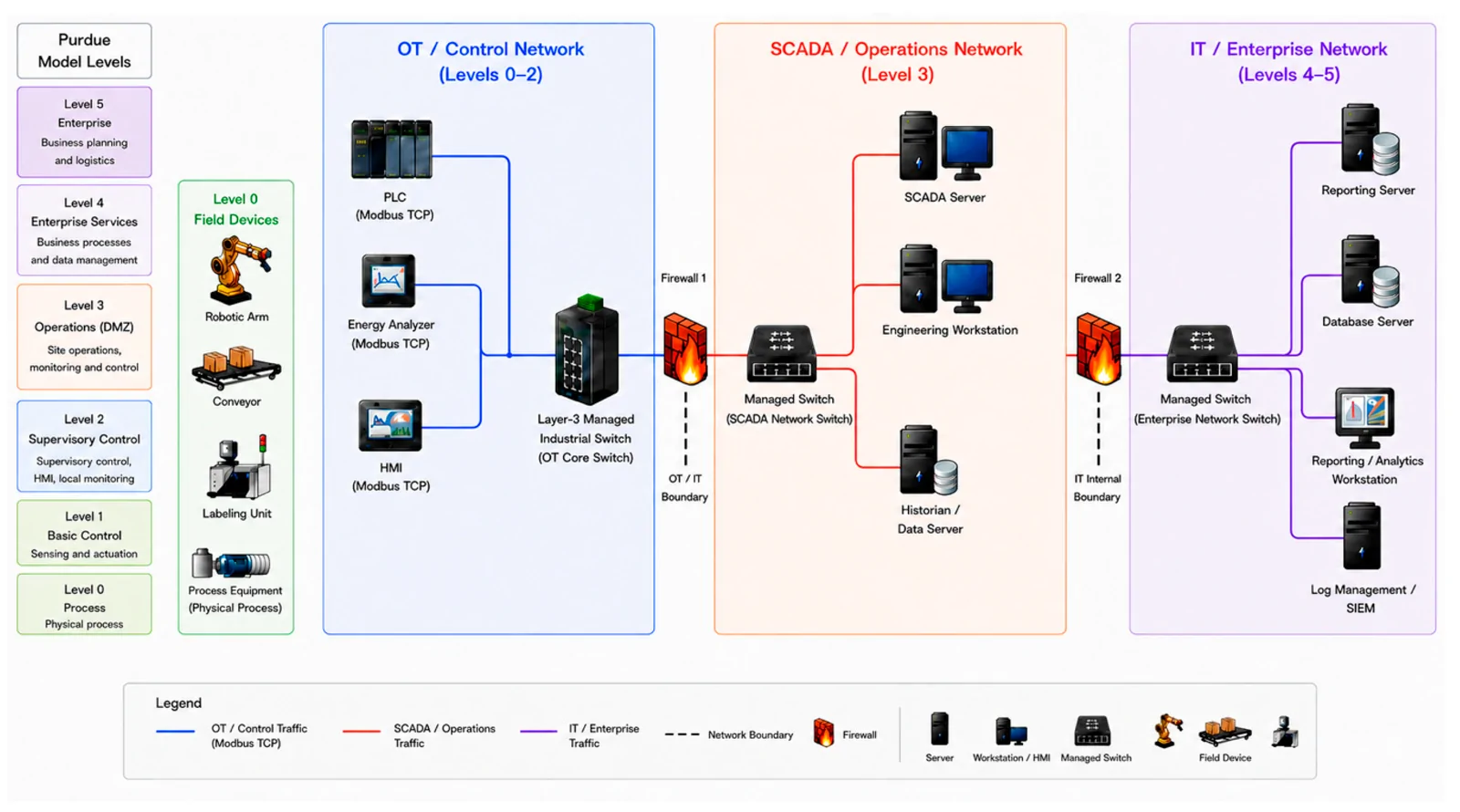
FALCON network architecture.

As illustrated in Figure 5, the FALCON testbed network architecture is designed in accordance with a layered approach that separates operational and enterprise domains. In the designed architecture, the OT layer is structured around an industrial-grade Layer-3 managed switch, which facilitates communication between core field devices, including the PLC, energy analyzer, and HMI. This switch acts as the primary aggregation point for OT traffic and enables controlled routing and segmentation. In terms of the Purdue Enterprise Reference Architecture [2], these components correspond to Levels 0–2, where Level 0 represents the physical process, Level 1 includes sensing and actuation devices, and Level 2 hosts local supervisory control such as HMI systems.

Secure interaction with upper layers is achieved by forwarding OT traffic through a software-based firewall implemented using pfSense (2.8 version) [55]. This transition marks the boundary between OT and IT domains (Level 3), where an intermediate network segment is established via a secondary managed switch. Within this segment, supervisory systems such as SCADA servers and other industrial applications are deployed, providing centralized monitoring and control.

A second firewall, also implemented using pfSense, is deployed between the Level 3 network and upper IT layers (Levels 4–5) to enforce additional segmentation. Beyond this boundary, the other switch hosts enterprise-level services, including reporting, logging, and data analytics systems.

The architecture has been designed to be multi-layered, in alignment with the Purdue model. This results in the enforcement of strict network boundaries, the minimisation of lateral movement, and the enablement of fine-grained monitoring across functional zones. This structured separation also enhances the testbed’s suitability for cybersecurity experimentation, including intrusion detection, traffic classification, and dataset generation.

## 5. Security Analysis on FALCON

The proposed FALCON testbed was designed not only as a physical representation of an industrial production environment, but also as an experimental platform for analyzing cyber-physical security risks in ICS/OT systems. In many conventional ICS testbed studies, security analysis is often limited to network-level attacks, traffic monitoring, or isolated control-system scenarios. In real industrial environments, however, the significance of an attack is determined by its technical compromise as well as by its impact on the physical production process.

Accordingly, the security analysis of the FALCON testbed is structured around two complementary attack perspectives: network-based attacks and process-based attacks. Network-based attacks target the communication, control, and monitoring layers, whereas process-based attacks directly target physical production behavior, sensors, actuators, robotic arms, conveyors, and station-based operations. This dual perspective enables the proposed testbed to support the analysis of both immediate and delayed physical consequences of cyber and physical attacks.

The main objective of this section is to demonstrate how FALCON can be used to evaluate cyber-physical impacts, process-level deviations, and operational risks under controlled and repeatable experimental conditions.

### 5.1. Threat Model

The threat model adopted in this study was developed by considering the layered network architecture of the FALCON testbed shown in Figure 5 and the process state diagram presented in Figure 2. The network architecture consists of the Level 0 physical process and field devices, the Levels 0–2 OT/Control Network, the Level 3 SCADA/Operations Network, and the Levels 4–5 IT/Enterprise Network. This structure enables the security analysis to be framed through the relationships among the physical process, control network, operational monitoring layer, and enterprise network, rather than through individual devices or isolated communication channels.

The threat model is based on two main attacker perspectives. The first perspective represents a cyber attacker with access to one of the network or system layers, such as the IT/Enterprise, SCADA/Operations, or OT/Control network. This attacker model is used to define the architectural context of the network-based attack scenarios discussed in the following sections. The second perspective represents an adversary who is physically close to the process or has process-aware capabilities. This model provides the basis for evaluating process-based attack scenarios associated with Level 0 components, including conveyors, robotic arms, sensors, actuators, and station-based production units.

The OT/Control Network–SCADA/Operations Network boundary and the SCADA/Operations Network–IT/Enterprise Network boundary shown in Figure 5 are considered as cross-layer access and propagation points in the threat model. These boundaries define the possible architectural position of an adversary. They also indicate the operational zones across which potential effects may propagate. In addition, the process state diagram in Figure 2 is used as a reference for assessing the possible position of attacks within the physical process flow. Accordingly, the threat model provides a process-oriented framework that considers product flow, process states, decision points, and the sequence of physical operations, in addition to the network layers.

The experimental assumptions are based on a scenario validation approach derived from the normal operating behavior of the FALCON testbed. In this approach, network-based and process-based scenarios are positioned and evaluated with respect to the relevant architectural layers and process states. Each scenario is therefore associated with both the layered network architecture and the physical process flow, providing a systematic basis for cyber-physical security analysis.

### 5.2. Network-Based Attack Scenarios

Network-based attack scenarios were structured to evaluate how cyber threats defined within the layered network architecture of the FALCON testbed can be associated with process-level effects. These scenarios were positioned according to the OT/Control Network, SCADA/Operations Network, and IT/Enterprise Network layers shown in the FALCON network architecture. This mapping links each attack scenario to a specific architectural zone, communication path, or operational function within the testbed.

The MITRE ATT&CK for ICS matrix [56] was used as the reference framework for classifying the scenarios. MITRE ATT&CK for ICS provides a structured knowledge base of adversarial behaviors targeting industrial control systems in terms of tactics and techniques. Accordingly, the network-based scenarios considered in this study were aligned with MITRE ATT&CK for ICS to maintain consistency with terminology commonly used in the ICS security literature.

In this study, the potential consequences of network-based attacks are represented at two complementary levels. The first level defines a generalized process-level impact category to improve the transferability of the analysis across ICS/OT environments. The second level identifies the corresponding FALCON-specific observable indicator, showing how the same impact can be associated with a physical process behavior in the proposed testbed. This two-level representation enables network-based attack scenarios to be interpreted not only from a network or device perspective, but also in relation to their process-level manifestations.

Table 2 summarizes the network-based attack scenarios in terms of their MITRE ATT&CK for ICS mapping, target layer in the FALCON architecture, expected cyber effect, generalized process-level impact, and FALCON-specific observable indicator.

The scenarios presented in Table 2 provide an architecture and process-oriented analysis framework for evaluating network-based attacks in the FALCON testbed. Each scenario is positioned according to its MITRE ATT&CK for ICS mapping, target layer in the FALCON network architecture, expected cyber effect, generalized process-level impact, and observable indicator in the testbed. This structure supports the interpretation of network-based attacks not only at the level of communication or device access, but also in relation to physical process behavior.

The scenarios are mainly considered through three impact domains: disruption of control communication, degradation of operational visibility, and manipulation of process data. Adversary-in-the-Middle scenarios targeting PLC-HMI, PLC-SCADA, and PLC-PLC communication are used to evaluate how manipulations along different communication paths may affect operator visibility or inter-unit coordination. Similarly, DoS-based scenarios are considered separately for control and monitoring communication, enabling the process-level implications of control loss and visibility loss to be distinguished.

Unauthorized command message and false data injection scenarios are positioned as representative cases for evaluating the potential propagation of network-based attacks to the physical process. In these scenarios, the analysis does not focus solely on whether unauthorized command transmission or data manipulation occurs. It also examines how such conditions can be associated with physical process indicators such as process sequence, actuation behavior, quality control decisions, or product flow.

HMI/SCADA view manipulation and SCADA visibility manipulation scenarios are evaluated in terms of operator awareness and process visibility. These scenarios are relevant for analyzing inconsistencies between the physical process and its digital representation, particularly in relation to delayed intervention, incorrect decision-making, or late detection of abnormal conditions. The lateral movement scenario from the IT/Enterprise Network toward the SCADA/Operations Network is evaluated primarily in terms of cross-layer propagation and potential access to the operational network, rather than direct physical process impact.

This framework provides a systematic basis for scenario-based validation of network-based attacks in the FALCON testbed. Accordingly, each attack scenario can be analyzed together with its architectural position in the network and its potential observable counterpart within the physical process flow.

### 5.3. Process-Based Attack Scenarios

Process-based attack scenarios were defined to evaluate process-level consequences of interventions that may directly affect the physical production process in the FALCON testbed. While network-based attack scenarios focus mainly on communication, control traffic, and digital visibility, process-based attack scenarios target field components, product flow, and station-based operations located in the Level 0 physical process area. Therefore, this section provides a complementary analysis layer that links the physically observable production behavior of FALCON with cyber-physical security assessment.

The process state diagram was considered when defining these scenarios. Accordingly, each attack scenario was evaluated not only according to the targeted physical component, but also in terms of its position within the process flow, decision points, and potential influence on the production sequence. The physical structure of the FALCON testbed, which includes conveyors, robotic arms, sensors, actuators, a linear axis, a quality control unit, labeling, and stamping stations, enables such scenarios to be analyzed under controlled and observable conditions.

In this study, process-based attack scenarios are represented at two complementary levels. The first level defines a generalized process-level impact category, which supports the transferability of the analysis to other smart manufacturing or ICS/OT environments. The second level identifies the corresponding observable indicator in the FALCON testbed, showing how the same impact can be associated with a physical behavior or process deviation. This structure enables physical interventions to be evaluated not only at the component level, but also in relation to process continuity, process integrity, product quality, and operational safety.

Table 3 summarizes the process-based attack scenarios in terms of the target component or process stage, physical intervention type, process-level impact, and observable indicator in FALCON.

The scenarios presented in Table 3 define representative intervention types that can be used to evaluate physically observable security conditions in the FALCON testbed. These scenarios are mainly assessed through sensor behavior, product flow, robotic arm movements, actuator triggering, quality control decisions, and station-based operation sequences. Accordingly, the process-based attack analysis focuses on process-level deviations that may affect the integrity of the production flow rather than treating each intervention as an isolated component failure.

The scenarios can be interpreted through four main impact domains. The first domain is the degradation of sensing and decision-making processes. Sensor blocking, sensor spoofing, and cable or connector tampering may create inconsistencies between the actual physical state of the product and the signal or detection information received by the control system. Such conditions can be associated with process-level deviations, such as the failure to initiate an operation when a product is present or the triggering of actuation when the expected product state is absent.

The second domain is the disruption of product flow and process sequencing. Conveyor timing manipulation, material flow obstruction, product misplacement, and process sequence manipulation may prevent the product from reaching the expected process stage at the expected time, position, or order. These deviations can be assessed by comparing the observed production flow with the process state diagram.

The third domain is deviation in physical actuation and handling behavior. Unauthorized actuator triggering, robotic arm misoperation, vacuum gripping degradation, pneumatic pressure manipulation, and linear axis obstruction may cause physical movements to deviate from the expected behavior in terms of timing, force, position, or sequence. In FALCON, these effects can be associated with observable indicators such as failed product pickup, incorrect robotic arm timing, unsuccessful product transfer, or the inability of the linear axis to reach the required station.

The fourth domain is product quality, traceability, and operational safety. Quality control bypass, labeling manipulation, stamping manipulation, product substitution, and emergency stop or safety interlock misuse are considered intervention types that may affect not only production continuity but also product correctness, traceability, and safe operating conditions. These scenarios are particularly useful for evaluating physical interventions that may produce meaningful consequences even without stopping the production line.

This framework provides a systematic basis for scenario-based validation of process-based attacks in the FALCON testbed. Each scenario is considered together with its target physical component, position in the process flow, generalized process-level impact, and observable indicator in the testbed. Therefore, FALCON can be positioned not only as a platform for evaluating the physical implications of network-based attacks, but also as an experimental environment for analyzing the effects of direct physical interventions on production behavior.

## 6. Result

This section presents the comparative results of the proposed testbed with respect to existing ICS cybersecurity testbeds. The evaluation is organized around two complementary perspectives. First, the design-level capabilities of the testbeds are compared in terms of architectural and functional requirements. Second, the experimental coverage of representative cyber and cyber-physical attack scenarios is analyzed. This two-tier evaluation enables the proposed testbed to be assessed as both an architectural platform and an experimental environment capable of supporting realistic security analysis.

### 6.1. Design Requirements

The architectural capability of the proposed testbed was evaluated by comparing it with existing ICS cybersecurity testbeds. The evaluation was based on key design requirements that define the practical and operational value of an experimental platform. These requirements help determine the extent to which each testbed can support realistic security experiments, reproducible analyses, scalable configurations, detailed process representation, and safe attack execution under controlled conditions.

The comparison considers eleven design requirements. Cost-effectiveness refers to the ability to build and operate a testbed within a reasonable budget while still meeting the intended research objectives and scenarios. Monitoring and logging capabilities enable the observation, recording, and subsequent analysis of process events. Flexibility refers to the ability to redefine or adapt the testbed for alternative usage scenarios. Reproducibility indicates whether testbeds built with the same design can produce comparable outputs. High fidelity reflects how closely the simulation, process behavior, and obtained results correspond to real-world observations. Scalability describes the ability to increase the size or functionality of the testbed. Granularity represents the level of detail used to model real-world processes and components. Isolation indicates whether cybersecurity scenarios and attack vectors can be executed in a safe environment without affecting external networks. Usability refers to the ease with which operators with reasonable technical skills can use the testbed. Diversity reflects the ability to incorporate different components without compromising scalability. Finally, interoperability refers to the ability of physical processes, network components, security mechanisms, and field devices to communicate and operate together toward the intended experimental goals.

Table 4 presents the comparative assessment of these design requirements across the reviewed testbeds. In this table, a positive mark (✓) indicates that the requirement is directly satisfied, a partial mark (△) indicates that the requirement is partially satisfied or supported in a limited manner, and an absence mark (X) indicates that the requirement is not meaningfully supported by the corresponding testbed architecture. The purpose of this comparison is to define the key design requirements of ICS cybersecurity testbeds and to examine the extent to which each platform supports the architectural and operational capabilities needed for comprehensive experimentation. Therefore, the aim is to provide a structured basis for understanding their design strengths, limitations, and experimental coverage. This evaluation also helps position the proposed testbed in relation to existing platforms and identify the specific design aspects in which it offers broader support.

The comparison in Table 4 highlights the trade-off between equipment cost and physical process representation in ICS testbeds. Software-based environments reduce hardware requirements, while physical implementations require investment in process and control equipment. FALCON partially satisfies cost-effectiveness because its design prioritizes physical process representation over minimizing cost. Nevertheless, more affordable device options were selected to limit the overall system cost.

High fidelity and granularity are supported by the integration of scaled factory processes, industrial control equipment, and realistic communication paths. FALCON was developed from observations of real industrial operations and includes PLC-to-PLC, PLC-to-SCADA, and PLC-to-HMI communication. This configuration allows experiments to capture controller-related delays, network latency, physical process delays, and process faults. Representing individual operations and their physical dependencies also allows cyber events to be examined alongside their effects on control actions and process behavior.

Monitoring and logging provide complementary views of these interactions. Network traffic is captured through switch port mirroring, while SCADA and other logging tools record events. Sensor measurements and process states are also recorded to support subsequent analysis of cyber and physical incidents. Isolation is addressed by physically separating FALCON’s network from the host institution’s network. When external connectivity is required, access is provided through a proxy behind a firewall configured with whitelist-based rules.

Flexibility is supported by combining physical processes with factory-like network infrastructure. This allows the platform to accommodate studies of device integration, preliminary predictive maintenance, potential attack targets, security impacts, and possible physical damage following cyber incidents. Scalability is addressed through the layered network structure, which can be extended with additional devices.

The reproducibility rating is based on the intended consistency of process flows, individual operations, and stage transitions with the represented factory workflow. This provides a design-based rationale for the assessment, without establishing repeatability across experimental runs. Usability is addressed through SCADA and HMI interfaces developed with usability principles. These interfaces support operator interaction with the process and aim to provide an accessible, realistic operating environment.

Diversity and interoperability are addressed through the integration of heterogeneous IT and OT technologies. The control, monitoring, and network infrastructure includes components from different manufacturers, while the virtualized IT environment can support different products, services, and operational configurations. The diversity rating therefore reflects component and vendor variety. Interoperability is supported by data exchange between PLCs from different manufacturers and an HMI supplied by another manufacturer, together with communication between the virtualized SCADA system and OT-layer PLCs. Applications and services can also access the OT layer through virtual networks within the layered security architecture, allowing physical devices, virtual services, and supervisory systems to operate within a common experimental environment.

FALCON offers a useful balance between realism and experimental coverage. Rather than being designed as a minimal-cost simulation environment, it aims to support cyber-physical experimentation through the integration of physical process execution, control components, supervisory monitoring, network segmentation, and security analysis within the same architecture.

### 6.2. Attack Scenario

Beyond design-level requirements, the practical utility of an ICS testbed is defined by the number of attack scenarios that can be executed and observed in the environment. Therefore, a second comparison was conducted by mapping representative attack scenarios to the capabilities of each reviewed testbed. The selected scenarios cover both network-oriented and process-oriented threats, including reconnaissance, adversary-in-the-middle attacks, denial-of-service conditions, unauthorized command transmission, HMI/SCADA manipulation, false data injection, visibility degradation, lateral movement, sensing manipulation, actuation manipulation, process sequencing manipulation, and safety/interlock misuse.

Table 5 summarizes the attack scenario coverage of the reviewed testbeds. In this table, a positive mark (✓) indicates that the reported testbed architecture supports execution of the attack scenario without architectural extensions, regardless of whether its execution was demonstrated in the corresponding study. A partial mark (△) indicates that the scenario could be supported through limited extensions to the reported architecture, such as additional components, software modules, or communication interfaces. An absence mark (X) indicates that supporting the scenario would require major architectural changes rather than limited extensions. The comparison shows that several existing studies focus on specific attack categories, such as DoS attacks, false data injection, process-aware manipulation, or operator-view deception. However, only a limited number of testbeds provide broad support across both cyber and physical dimensions of ICS security. FALCON supports all evaluated attack scenarios at the architectural level and provides an environment for systematic cyber-physical security experimentation.

As shown in Table 5, FALCON provides broader attack-scenario coverage than the reviewed studies. Many existing testbeds mainly focus on network-based attacks or related security tasks, such as denial-of-service attacks, false data injection, adversary-in-the-middle attacks, intrusion detection, and training-oriented exercises. In contrast, FALCON supports a wider set of real-world scenarios by covering network-oriented attacks, process-oriented manipulations, and the observable physical consequences of cyber-attacks.

This broad coverage is mainly enabled by the integration of physical process components, PLC-level control, HMI/SCADA supervision, controller-to-controller communication, monitoring channels, and IT–OT network segmentation within the same experimental environment. The comparison considers whether each testbed includes the components and communication interfaces required to carry out each attack. Some of the compared testbeds lack components needed for particular scenarios, so these attacks cannot be performed using their reported configurations. A partial mark (△) is assigned when the missing components can be added without major architectural changes. FALCON receives a positive mark (✓) because its existing infrastructure includes the components and interfaces required for the corresponding scenarios, including those not experimentally demonstrated in this study. These positive marks therefore indicate architectural support and should not be interpreted as experimental validation of every scenario. Therefore, the testbed is not limited to executing network attacks and observing their effects only at the network layer. It can also be used to analyze how a threat originating from any layer may propagate across the cyber, control, monitoring, and physical process layers. This capability makes the testbed suitable not only for evaluating individual attacks, but also for analyzing multi-stage attack chains involving discovery, lateral movement, communication interception, command manipulation, process-value falsification, and operator-view deception. FALCON offers a flexible and realistic environment for validating cyber-physical security scenarios under controlled laboratory conditions. More importantly, its architectural support for all attack scenarios compared in the table strengthens its suitability for systematic threat modeling, attack-impact analysis, and the development of detection and mitigation mechanisms for ICS/SCADA environments.

### 6.3. Experimental Validation

This section presents the experimental implementation and observes effects of selected attack scenarios described in Section 5. FALCON’s normal operation is first described using the accompanying figure and process data. This establishes a reference for evaluating the changes caused by the attacks. The subsequent subsections examine network-based and process-based attacks, comparing the behavior of the affected components under normal operation and attack conditions to assess the effects on control functions and the physical process.

**Figure 6:**
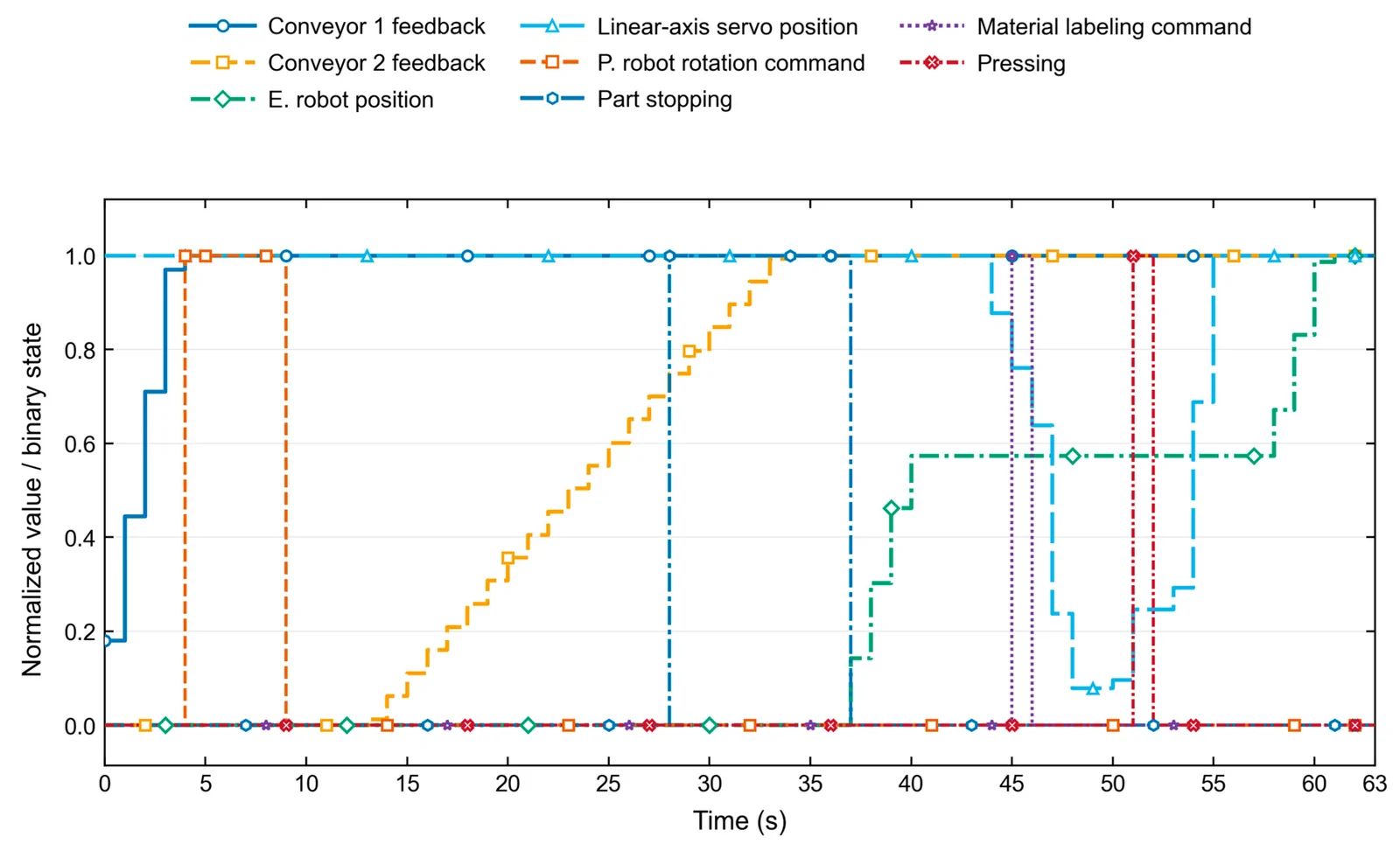
Single production cycle in FALCON under normal operating conditions.

Figure 6 shows the sequence of operations involved in transporting and processing raw material during a single production cycle under normal operating conditions.

Conveyor-1 completes its initial movement at approximately 4 s, after which the P-robot rotation command is activated. The P-robot turns towards Conveyor-1, retrieves the raw material, and rotates back to place it on Conveyor 2. Conveyor-2 starts moving at approximately 13 s, and the product reaches its downstream end at approximately 33 s. Detection by the sensor at the end of the conveyor activates the stopper mechanism, retaining the product at the transfer point. The stopper command remains active from approximately 28 to 37 s, overlapping the final portion of the Conveyor-2 movement.

Once Conveyor-2 completes its movement, the E-robot retrieves the product and places it on the linear axis. This transfer corresponds to the change observed in the E-robot position signal between approximately 37 and 40 s. The linear axis transports the product sequentially to the labeling and stamping stations before returning it to the E-robot pickup position. The linear-axis position signal changes between approximately 44 and 55 s. The labeling command is active at approximately 45–46 s, followed by the pressing command associated with stamping at approximately 51–52 s.

The E-robot position remains constant throughout the operation of the linear axis. After the product returns to the pickup position, the E-robot resumes movement at approximately 58 s, retrieves the processed product, and places it back on Conveyor-1. Its final movement is completed at approximately 62 s. These traces provide the reference operating sequence for assessing changes in component behavior and process timing under attack conditions.

#### 6.3.1. Network-Based Attack

The network-based attack is evaluated under an insider threat model in which the attacker has access to the network switch serving OT/control network at Levels 0–2 (Figure 5). Through this access, the attacker communicates with PLC-1 via Modbus TCP and issues unauthorized coil write commands. The attack falls within the “Unauthorized Command Message” category listed in Table 2 and targets Modbus coil 1299, which corresponds to the stopper mechanism controlled through PLC-1’s expansion module.

The attack is triggered by the operating state of the stopper mechanism. The attacker monitors the target coil over the network and waits for its value to become 1, indicating that the mechanism is active. Upon detecting this value, the attacker writes 0 to the same coil to deactivate the mechanism. A subsequent write of 1 reactivates it. This sequence allows the attacker to manipulate the activation and deactivation of the stopper through unauthorized write operations over the network.

**Figure 7:**
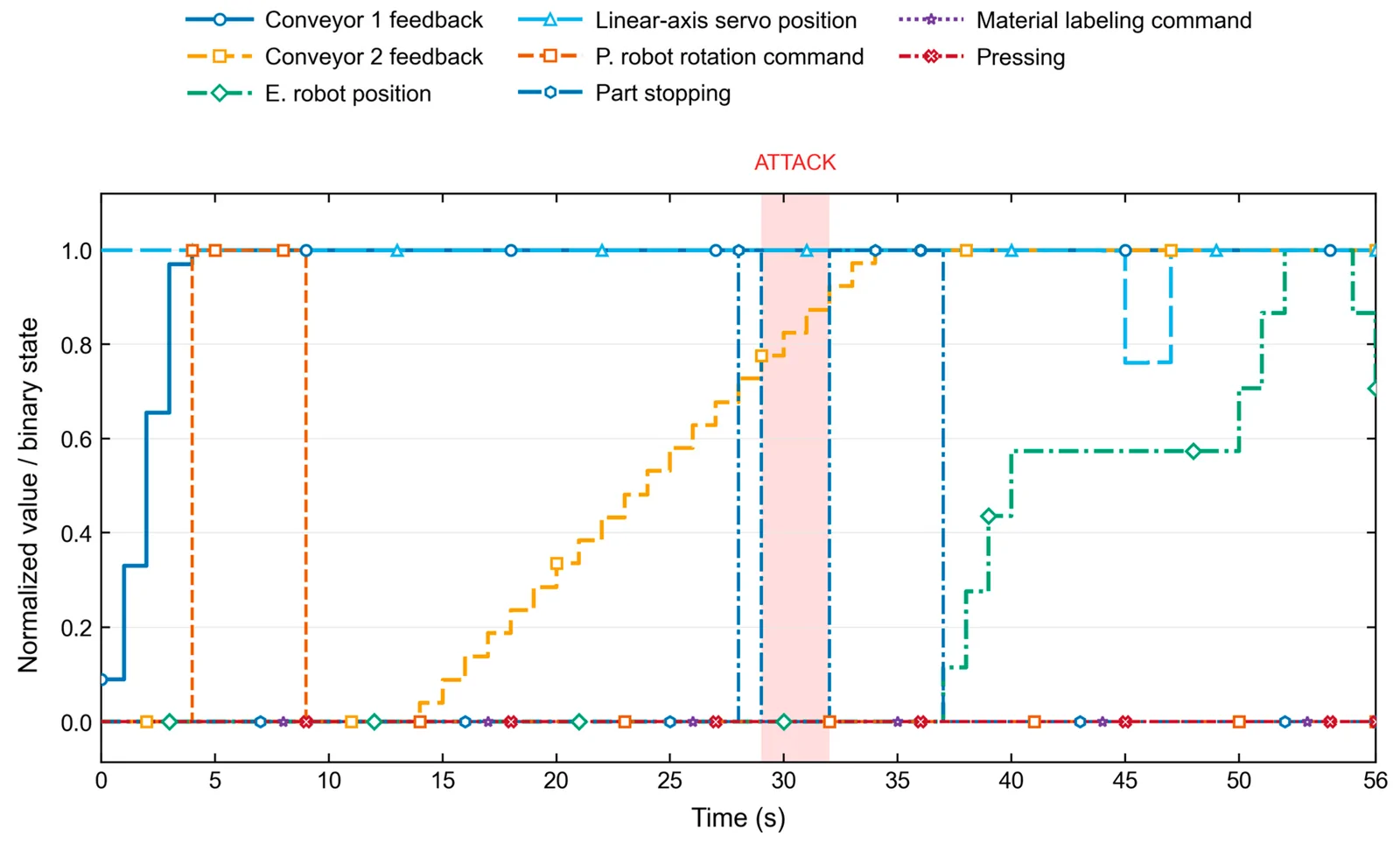
Single production cycle in FALCON under network-based attack conditions.

Figure 7 shows the behavior of the components during network-based manipulation of the stopper mechanism. The red shaded region in the figure marks the attack interval. 

The process follows the normal operating sequence until the product is detected by the sensor at the end of Conveyor-2. The attacker monitors the mechanism’s state over the network, waits for its activation, and then disables it by writing to the corresponding coil via Modbus TCP. The stopper command becomes active at approximately 28 s but is forced into an inactive state by the attacker between approximately 29 and 32 s. During this interval, the continued increase in the Conveyor-2 position signal indicates that the transport movement is maintained. As observed during the experiment, disabling the stopper while the conveyor remains in motion causes the product to fall off Conveyor-2.

After the product falls, the stopper mechanism is reactivated at approximately 32 s, and the command remains active until approximately 37 s. Within the implemented control logic, this intervention re-establishes the condition indicating that the product is ready for transfer. The change in the E-robot position signal between approximately 37 and 40 s shows that the pickup and placement sequence observed during normal operation is executed despite the absence of a product at the transfer point. E-robot therefore continues its motion sequence without transferring a physical product.

A brief change in the linear-axis position signal is subsequently observed between approximately 45 and 47 s. Experimental observations indicate that, because no product is present, the color inspection cannot identify a valid product, preventing progression to the labeling and stamping stages. Consistent with this observation, the labeling and pressing commands that become active during normal operation remain inactive throughout the attack recording. The E-robot position remains constant during this linear-axis movement, and the position changes beginning at approximately 50 s indicate the initiation of the next transfer step. The robot executes the motions associated with retrieving the product from the linear axis and returning it to Conveyor-1, although no product is present.

In this experiment, temporarily disabling the stopper causes product loss, while reactivating it allows the control sequence to continue in a manner inconsistent with the physical presence of the product. The subsequent color check prevents progression to labeling and stamping but does not prevent the E-robot from executing handling motions without a product. This behavior reveals a discrepancy between the logical transfer-ready condition and the actual physical state of the product.

#### 6.3.2. Process-Based Attack

In the process-based attack scenario, the attacker is modeled as a local adversary with physical access to the compressed-air supply serving FALCON pneumatic system. This access allows the attacker to shut off the supply without authorization. The attack targets the compressed air required to operate the pneumatic actuators.

During a production cycle, the attacker shuts off the supply, cutting the airflow into the targeted pneumatic circuit. The objective is to prevent the pneumatic components from performing their expected movements and disrupt the production sequence.

**Figure 8:**
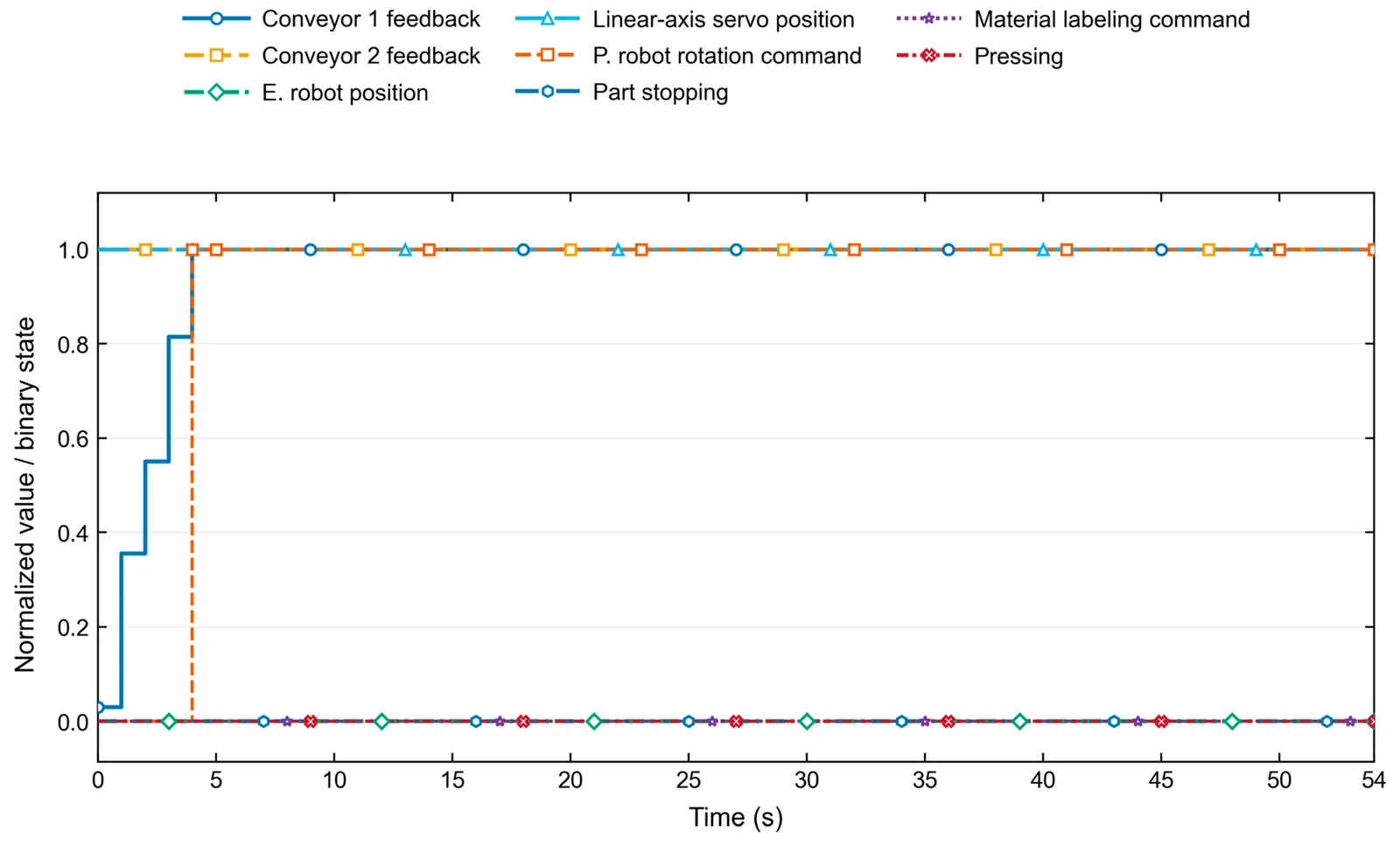
Single production cycle in FALCON under process-based attack conditions.

Figure 8 shows the behavior of the production cycle during a physical attack on the main compressed-air supply line. Conveyor-1 completes its initial transport movement at approximately 4 s, after which the P-robot rotation command becomes active. During normal operation, this command is active for a limited period. Under attack conditions, however, it remains active until the recording ends at 54 s. Physical observations during the experiment show that the loss of air supply prevents the P-robot from moving towards Conveyor-1, picking up the raw material, and transferring it to Conveyor-2. The control command is therefore issued, but the expected pneumatic movement does not occur.

The failure to complete the initial material transfer prevents the subsequent sequence from starting with Conveyor-2. Its feedback signal remains constant at 1 throughout the recording, with no change associated with a new transport movement. Similarly, the E-robot position signal remains at 0, while the linear-axis position signal remains at 1. The stopper, labeling, and pressing commands also remain inactive throughout the observation period. These signals show that the transfer and processing stages that normally follow one another are not initiated in this experiment.

The first observed effect of the air supply interruption is the P-robot inability to transfer the raw material. This interruption prevents the conditions required for the downstream stages from being met, leaving the production cycle unable to progress during the recording. The sustained rotation command and the absence of physical movement reveal a mismatch between the control command and the actuator response, illustrating the role of the pneumatic energy supply in executing the commanded motion.

## 7. Conclusions

This study presented FALCON, a cyber-physical ICS/SCADA testbed designed to support realistic, repeatable, and controlled cybersecurity experimentation. The proposed testbed integrates a physical production process, PLC-based control, HMI/SCADA supervision, monitoring and logging capabilities, IT–OT network segmentation, and process-level observability within a single experimental environment. This integrated structure allows cyber events to be examined together with their effects on control behavior, operator visibility, and physical process outcomes.

The results were evaluated from two complementary perspectives. First, the design requirement comparison showed that FALCON provides broad architectural support across the evaluated criteria. Although cost-effectiveness was partially satisfied due to the use of real physical process components and industrial control devices, this design choice increased the realism and experimental value of the testbed. FALCON fully satisfied the remaining requirements, including monitoring and logging, flexibility, reproducibility, high fidelity, scalability, granularity, isolation, usability, diversity, and interoperability. This indicates that the proposed architecture offers a balanced design between practical implementation cost and cyber-physical experimentation capability.

Second, the attack scenario comparison supported that FALCON supports a wide range of network-based and process-based attack scenarios. Unlike several existing testbeds that mainly focus on specific attack categories, such as denial-of-service, false data injection, adversary-in-the-middle attacks, or intrusion detection, FALCON enables the analysis of multi-layer attack behavior. The testbed supports scenarios involving network and device discovery, communication interception, unauthorized command transmission, HMI/SCADA view manipulation, false process-value injection, SCADA visibility manipulation, lateral movement, sensing manipulation, actuation manipulation, process sequencing manipulation, and safety/interlock misuse. This coverage makes it possible to evaluate not only isolated attacks, but also multi-stage attack chains that propagate across cyber, control, monitoring, and physical process layers.

The main value of FALCON lies in its ability to connect cyber-layer activities with observable physical consequences. By combining real process execution with industrial control and supervisory components, the testbed provides a suitable environment for threat modeling, attack-impact analysis, anomaly detection, and the validation of mitigation strategies. This capability is particularly important for ICS/SCADA cybersecurity research, where the severity of an attack is often determined not only by its network behavior, but also by its operational and physical effects.

FALCON is a laboratory-scale testbed and cannot fully represent the size, complexity, and operational constraints of large industrial facilities. In addition, the current implementation focuses on a specific production-line process, and future extensions may be needed to represent other industrial domains such as energy, water treatment, or chemical processes. Future work will focus on incorporating additional industrial protocols, extending attack scenarios to other application domains, automating scenario execution, developing advanced intrusion detection mechanisms, and integrating digital twins. These extensions will further improve the ability of FALCON to support comprehensive and reproducible ICS/SCADA cybersecurity research under controlled laboratory conditions.

## Figures and Tables

**Table 1:** Comparison of the reviewed digital-twin studies and ICS cybersecurity testbeds.

Study	Approach	Application Domain	Reported Security Focus
Olivares-Rojas et al. [12]	Digital twin	Smart-home advanced metering infrastructure	Vulnerability assessment in a virtual representation of smart-metering systems.
Masi et al. [13]	Cybersecurity-oriented digital twin	Critical infrastructure and industrial automation	Security-driven modelling, attack-path analysis, and evaluation of countermeasures.
Holmes et al. [14]; Repetto [15]	Digital-twin security analysis	Digital representations of physical infrastructure	Additional attack surfaces and the potential use of digital twins for adversarial intelligence gathering.
EPICTWIN [16,17]	Digital twin of a physical testbed	Electric power systems	Protocol-level interactions, attack scenarios, and cybersecurity assessment using a virtual counterpart of EPIC.
Queiroz et al./SCADASim [19]	Software-based simulation using OMNeT++	SCADA systems	Evaluation of DDoS and adversary-in-the-middle scenarios using simulated control and communication components.
Antonioli et al./MiniCPS [20]	Software-based simulation and network emulation	Water treatment and generic CPS applications	Investigation of industrial communications, control interactions, and attack and defence scenarios.
Morris et al. [21]	Virtual testbed	Gas pipeline	Sensor attacks and control-state analysis using simulated PLC, HMI, and process components.
Formby et al./GRFICS [22]	Virtual ICS with 3D process visualization	Chemical process	Security training and visualization of the effects of command injection and buffer-overflow attacks.
Maynard et al. [23]	VM-based SCADA framework with scripted deployment	Generic SCADA networks	Configurable HMI–RTU communication and repeatable deployment of experimental network environments.
Gao et al./EPS-ICS [24]	Real PLCs with HIL process simulation and network emulation	Industrial control systems	PLC vulnerability analysis within an integrated control, process, and communication environment.
Koganti et al. [25]	Simulated control/process components with physical switches	Industrial control networks	Flexible experimentation through configurable simulation scripts and a physical network layer.
Faramondi et al. [26]	HIL water process integrated with virtual ICS components	Water distribution	Cyber-physical attack evaluation and collection of normal and attack-state data.
Mallouhi et al. [27]	Integration of physical and virtual components	SCADA and control-system environments	Real-time event simulation and interactions between physical and virtual elements.
Koutsandria et al. [28]	Real PLCs, simulated process, and network taps	Simulated OT process	Attack experimentation and monitoring of network statistics and packet-level changes.
Keliris et al. [29]	Hybrid hardware–software platform	Industrial control systems	Vulnerability and impact analysis, including evaluation of machine learning-based defences.
Ghaleb et al./SCADA-SST [30]	Simulation platform supporting hybrid configurations	SCADA applications	Communication between simulated and physical devices and assessment of DoS resilience.
Xie et al./VTET [31]	Virtualization and process simulation	Tennessee Eastman chemical process	ICS protocol interactions and cybersecurity experimentation within a simulated process environment.
Mathur et al./SWaT [32]	Physical testbed	Six-stage water treatment	Multi-stage cyber-physical attacks and observation of their effects on a real treatment process.
National SCADA Testbed [33]	Field-scale physical infrastructure	Electric power distribution	Cyber-resilience assessment of energy control systems using security scenarios and modelling tools.
Alves et al./OpenPLC [34]	Open-source controller platform with physical implementations	Industrial control applications	Accessible, low-cost implementation of hardware-based control experiments.
Sauer et al./LICSTER [35]	Low-cost physical testbed	Small-scale industrial control process	Practical security experimentation and education using open-source components.
Adepu et al./EPIC [17]	Physical testbed	Smart grid	Evaluation of cyberattacks and their physical consequences using real controllers and industrial communications.
FALCON/This study	Physical, laboratory-scale ICS/SCADA testbed	Multi-stage manufacturing with conveyors, robotic transfer, and quality control	Structured network- and process-based attack analysis, supported by two representative experiments and recorded process-state comparisons.

**Table 2:** Network-based attack scenarios on FALCON.

Attack Scenario	MITRE ATT&CK for ICS Mapping	Target Layer/Corresponding Component in the FALCON Architecture	Cyber Effect	Process-Level Impact	Observable Indicator in FALCON
Network and device discovery	Remote System Information Discovery; Network Connection Enumeration	OT/Control Network (Levels 0–2): PLC, HMI, Energy Analyzer, Layer-3 Managed Industrial Switch	Identification of active devices, services, protocols, and communication relationships	No direct process-level effect is expected; it serves as a preparatory step for subsequent targeted attacks	Mapping of PLC, HMI, SCADA, switch, and Modbus TCP communication flows
Adversary-in-the-Middle on PLC-HMI communication	Adversary-in-the-Middle; Manipulation of View	OT/Control Network (Levels 0–2): PLC–HMI Modbus TCP communication	Monitoring, delaying, or manipulating process data transmitted to the HMI	Loss of operator visibility; delayed response	The operator observes conveyor, robotic arm, or station status incorrectly or with delay on the HMI
Adversary-in-the-Middle on PLC-SCADA communication	Adversary-in-the-Middle; Manipulation of View	OT/Control Network–SCADA/Operations Network boundary: PLC–Firewall 1–SCADA Server communication	Monitoring, delaying, or manipulating process data transmitted from the PLC to SCADA	Loss of situational awareness; delayed operational response	Inconsistency between the SCADA process view and the physical production line
Adversary-in-the-Middle on PLC-PLC communication	Adversary-in-the-Middle; Block Operational Technology Message; Manipulation of Control	OT/Control Network (Levels 0–2): PLC–PLC communication	Delaying, blocking, or modifying messages between control units	Inter-unit coordination loss; process desynchronization	Disruption of the operation sequence among the conveyor, robotic arm, and linear axis
DoS against control communication	Block Communications; Denial of Control; Block Operational Technology Message	OT/Control Network (Levels 0–2): PLC, OT Core Switch, Modbus TCP control traffic	Communication delay, blocked command transmission, or service interruption in control communication	Process disruption; delayed actuation	Conveyor stoppage, delayed robotic arm triggering, or interruption of product transfer
DoS against monitoring communication	Denial of View; Block Reporting Message	SCADA/Operations Network (Level 3): SCADA Server, Engineering Workstation, Historian/Data Server, SCADA Network Switch	Temporary or continuous unavailability of monitoring and operational data flow	Loss of process visibility; delayed operator response	The operator cannot observe the actual process state through SCADA or detects abnormal conditions with delay
Unauthorized command message	Unauthorized Command Message; Manipulation of Control	OT/Control Network (Levels 0–2): PLC control channel and Modbus TCP command traffic	Transmission or execution of unauthorized control commands	Unauthorized actuation; sequence violation	Robotic arm movement at an incorrect time, unexpected actuator triggering, or disruption of the process sequence
HMI/SCADA view or alarm manipulation	Manipulation of View; Denial of View	Level 2–Level 3 monitoring path: HMI, SCADA Server, Engineering Workstation	Modification of process status, alarm information, or operator visibility	Operator misperception; delayed response	Operator decision-making based on incorrect alarms, missing alarms, or misleading process status
False data injection into process values	Spoof Reporting Message; Manipulation of View; Modify Parameter	OT/Control Network and SCADA/Operations Network: PLC, HMI, SCADA Server, Historian/Data Server	Manipulation of sensor, tag, setpoint, or process values	Incorrect process decision; quality control violation	Bypassing quality control, routing the product to an incorrect station, or making an incorrect process decision
SCADA visibility manipulation	Manipulation of View; Denial of View	SCADA/Operations Network (Level 3): SCADA Server, Historian/Data Server, Engineering Workstation	Incorrect, incomplete, or delayed representation of the process state	Loss of situational awareness; delayed operational response	Inconsistency between the SCADA screen and the physical process
Lateral movement from IT to OT	Lateral Tool Transfer; Remote Services; Valid Accounts	IT/Enterprise Network to SCADA/Operations Network: Firewall 2, Enterprise Switch, SCADA Network	Unauthorized access or propagation from the IT layer toward the SCADA/Operations layer	Increased attack surface; potential operational exposure	Observation of transition attempts from Log/SIEM, reporting, or database systems toward the operational network

**Table 3:** Process-based attack scenarios and observable process indicators in FALCON.

Process-Based Attack Scenario	Target Component/Process Stage	Physical or Process-Level Intervention	Process-Level Impact	Observable Indicator in FALCON
Sensor blocking	Product detection, end-position, or raw-material sensor	Sensor field is physically blocked or covered	Missed detection; transition failure	Product is present, but the next conveyor, robotic arm, or station action is not triggered
Sensor spoofing	Raw-material, counting, or end-position sensor	False presence/absence information is physically induced	Incorrect process decision; premature or omitted actuation	Robotic arm or actuator is triggered without the expected product state
Conveyor timing manipulation	Conveyor-1 or Conveyor-2	Conveyor motion, speed, or timing is altered	Process desynchronization; delayed product transfer	Product reaches the next sensor or station earlier/later than expected
Unauthorized actuator triggering	Pneumatic or electromechanical actuator	Actuator is manually or locally triggered outside the expected sequence	Unauthorized actuation; sequence violation	Unexpected pushing, lifting, stamping, or transfer action occurs
Robotic arm misoperation	P-Robot or E-Robot	Robotic arm motion is physically obstructed, redirected, or forced out of sequence	Transfer failure; unsafe or incorrect handling	Product is not picked, dropped, misplaced, or transferred at the wrong time
Quality control bypass	Quality control unit	Product passes without the expected inspection condition being satisfied	Quality control violation	Defective or unverified product continues to the next process stage
Labeling manipulation	Labeling unit	Label is removed, misapplied, or incorrectly positioned	Traceability error; product identification failure	Product reaches the next stage with missing or incorrect label information
Stamping manipulation	Stamping unit	Stamp action is blocked, repeated, or applied to the wrong product position	Product integrity violation	Missing, duplicated, or misplaced stamp is observed
Product misplacement	Product holder, station area, or transfer point	Product is manually shifted from its expected position	Processing failure; mechanical mismatch	Robotic arm, linear axis, or station operation does not align with product position
Linear axis obstruction	Linear positioning system	Movement of the linear axis is physically blocked or restricted	Positioning failure; station sequence interruption	Product cannot reach the expected quality control, labeling, or stamping station
Vacuum gripping degradation	Robotic arm vacuum gripper	Vacuum suction is weakened, blocked, or leaked	Unreliable product handling; transfer failure	Robotic arm attempts to pick the product but drops it or fails to hold it
Pneumatic pressure manipulation	Pneumatic robotic arm or actuator line	Air pressure is reduced, interrupted, or made unstable	Delayed or incomplete actuation	P-Robot movement becomes weak, slow, incomplete, or inconsistent
Material flow obstruction	Conveyor path or transfer point	Product flow is physically blocked or congested	Production flow interruption; accumulation	Products accumulate before a sensor, transfer point, or station
Process sequence manipulation	Multi-stage production flow	A product is inserted, removed, or reordered during the process	Sequence violation; traceability inconsistency	The observed physical product order differs from the expected process state diagram
Manual mode or local override misuse	PLC-connected local control, HMI, or field interface	Normal automatic sequence is interrupted through local/manual intervention	Control-mode inconsistency; unexpected operation state	Physical process behavior differs from the automatic sequence expected by SCADA/HMI
Emergency stop or safety interlock misuse	Emergency stop, safety relay, or interlock point	Safety-related input is triggered or prevented outside expected conditions	Production interruption; safety-state inconsistency	System enters an unexpected stop state or fails to proceed to the next process step
Cable or connector tampering	Sensor, actuator, PLC I/O, or communication cable	Cable is loosened, disconnected, or intermittently disturbed	Signal loss; intermittent process failure	Unstable sensor readings, missed actuator response, or repeated process interruption
Product substitution	Raw-material input or station input	Expected product is replaced with a different or unsuitable product	Incorrect classification; quality or routing error	Product type, label, stamp, or quality decision does not match the expected process state

**Table 4:** Comparison of design requirements.

Design Requirement	TASSCS [28]	[57]	[29]	SCADA-SST [31]	HEDVa [58]	HYDRA [59]	VTET [32]	CANVAS [60]	SCASS [10]	HIL-TPP [61]	HINT-SEC [54]	ICSSIM [50]	[62]	[63]	[64]	[65]	FALCON
Cost-effectiveness	✓	✓	△	△	X	X	✓	△	△	△	△	✓	✓	△	△	✓	△
Monitoring and logging	✓	△	✓	△	✓	△	△	✓	✓	✓	✓	△	✓	△	X	✓	✓
Flexibility	△	△	△	△	△	△	△	△	✓	△	△	✓	△	✓	△	✓	✓
Reproducibility	✓	△	△	✓	X	✓	△	△	✓	△	✓	✓	△	✓	✓	△	✓
High Fidelity	X	X	X	X	△	△	X	X	✓	△	✓	X	X	△	△	△	✓
Scalability	X	X	X	△	△	△	△	△	✓	△	△	✓	X	X	X	△	✓
Granularity	X	X	X	△	△	X	△	△	△	X	✓	X	△	X	X	✓	✓
Isolation	✓	✓	✓	✓	✓	✓	✓	✓	✓	✓	✓	✓	△	X	X	✓	✓
Usability	△	△	△	△	△	△	X	✓	✓	△	△	✓	✓	△	△	✓	✓
Diversity	X	X	X	X	△	X	X	X	X	X	△	X	△	△	△	✓	△
Interoperability	X	X	△	X	X	X	X	△	△	X	✓	X	△	✓	X	✓	✓

**Table 5:** Comparison of attack scenarios.

Attack Scenarios	TASSCS [28]	[57]	[29]	SCADA-SST [31]	HEDVa [58]	HYDRA [59]	VTET [32]	CANVAS> [60]	SCASS [10]	HIL-TPP [61]	HINT-SEC [54]	ICSSIM [50]	[62]	[63]	[64]	[65]	FALCON
	**Network-based attacks**																
Network and device discovery	✓	X	△	△	✓	△	X	✓	△	✓	△	✓	△	△	✓	✓	✓
Adversary-in-the-Middle on PLC-HMI communication	✓	△	X	△	✓	✓	X	✓	✓	X	X	✓	△	X	✓	✓	✓
Adversary-in-the-Middle on PLC-SCADA communication	✓	△	X	△	X	X	X	X	X	✓	△	X	△	△	X	△	✓
Adversary-in-the-Middle on PLC-PLC communication	△	X	△	X	X	X	✓	X	△	X	△	△	X	△	X	△	✓
DoS against control communication	✓	X	△	✓	△	X	X	△	△	X	△	✓	✓	△	△	✓	✓
DoS against monitoring communication	✓	X	△	✓	△	X	X	△	△	✓	△	△	△	△	△	△	✓
Unauthorized command message	✓	△	△	△	△	△	✓	✓	△	✓	✓	✓	△	△	✓	✓	✓
HMI/SCADA view or alarm manipulation	✓	△	X	X	X	X	X	✓	✓	✓	△	△	✓	△	△	X	✓
False data injection into process values	✓	✓	△	X	△	△	✓	✓	✓	✓	✓	✓	△	△	✓	✓	✓
SCADA visibility manipulation	✓	△	△	△	X	X	X	△	△	✓	△	△	△	△	△	△	✓
Lateral movement from IT to OT	△	X	X	△	△	X	X	△	X	X	X	X	X	X	X	✓	✓
	**Process-based attacks**																
Sensing manipulation	△	△	△	X	△	△	✓	✓	X	✓	✓	✓	✓	X	✓	X	✓
Actuation manipulation	✓	X	△	△	△	△	X	△	X	X	X	X	✓	△	△	X	✓
Process sequencing manipulation	X	X	X	X	X	X	X	X	X	X	X	X	X	△	X	X	✓
Safety/interlock misuse	X	X	△	X	X	X	X	X	X	△	△	X	X	X	X	X	✓
Physical consequence of cyber attack	X	X	X	X	X	✓	X	X	X	X	X	X	✓	△	X	X	✓

## Data Availability

The experimental process data and network packet captures collected during normal operation and the network- and process-based attack experiments are provided in the Supplementary Materials.

## Supplementary Materials

The following supporting information can be downloaded at: https://www.mdpi.com/article/10.3390/s26216951/s1.

## Abbreviations

DoS	Denial of Service
FALCON	Factory Automation Lab for Cybersecurity & Operational Networks
HMI	Human–Machine Interface
ICS	Industrial Control System
IT	Information Technology
OT	Operational Technology
PLC	Programmable Logic Controller
SCADA	Supervisory Control and Data Acquisition
